# Extreme obesity induces massive beta cell expansion in mice through self-renewal and does not alter the beta cell lineage

**DOI:** 10.1007/s00125-016-3922-7

**Published:** 2016-03-22

**Authors:** Aaron R. Cox, Carol J. Lam, Matthew M. Rankin, Kourtney A. King, Pan Chen, Ramon Martinez, Changhong Li, Jake A. Kushner

**Affiliations:** Section of Pediatric Diabetes and Endocrinology, McNair Medical Institute, Baylor College of Medicine, Houston, TX 77030 USA; Diabetes and Endocrinology, Feigin Center, Texas Children’s Hospital, Houston, TX USA; Division of Endocrinology and Diabetes, Children’s Hospital of Philadelphia, Perelman School of Medicine, University of Pennsylvania, Philadelphia, PA USA; Diabetes and Endocrinology Service, Texas Children’s Hospital, Houston, TX USA

**Keywords:** Beta cell expansion, Cell fate, Lineage tracing, Obesity

## Abstract

**Aims/hypothesis:**

Understanding the developmental biology of beta cell regeneration is critical for developing new diabetes therapies. Obesity is a potent but poorly understood stimulus for beta cell expansion. Current models of obesity are complicated by developmental compensation or concurrent diabetes, limiting their usefulness for identifying the lineage mechanism(s) of beta cell expansion. We aimed to determine whether acute inducible obesity stimulates beta cell expansion and to determine the lineage mechanism of beta cell growth in obesity.

**Methods:**

We created whole-body tamoxifen-inducible leptin receptor (*LepR*)-deficient mice (*Ubc-Cre*^*ERT2*^*LepR*^*loxP/loxP*^) as a novel model of acute obesity. Beta cell mass and proliferation were quantified after short-term *LepR* deletion. Clonal analysis of beta cell expansion using the Brainbow2.1 reporter was performed 6 months post tamoxifen initiation.

**Results:**

*LepR* deficiency induced a doubling of body mass within 3 weeks, with moderate glucose intolerance (unlike typical *LepR* mutant mice [*db/db*], which have frank diabetes). Beta cell mass expanded threefold through increased beta cell proliferation, without evidence for contribution from specialised progenitors or stem cells (via sequential thymidine labelling and Brainbow2.1 reporter). Thus, self-renewal is the primary lineage mechanism in obesity-induced beta cell expansion. However, even the rapid beta cell proliferation could not exceed the restrictions of the replication refractory period.

**Conclusions/interpretation:**

In summary, we created a novel model of inducible obesity demonstrating that even extreme metabolic demand does not alter beta cell lineage.

**Electronic supplementary material:**

The online version of this article (doi:10.1007/s00125-016-3922-7) contains peer-reviewed but unedited supplementary material, which is available to authorised users.

## Introduction

Absolute or relative deficiency of beta cell mass underlies diabetes. Endogenous beta cell regeneration represents a promising approach to restore functional beta cell mass. However, adult beta cell turnover is minimal, with very low rates of beta cell proliferation and apoptosis [1–4]. Current efforts to stimulate human beta cell regeneration have proven futile [5–9], although obese individuals have profound beta cell expansion compared with lean individuals [4, 10]. Thus, obesity represents an exceptional model with which to study the mechanisms of beta cell regeneration.

Very little is known about the lineage mechanism of beta cell expansion in obesity. Butler et al observed an increase in insulin-positive cells associated with exocrine ducts [10]. Given the low rates of beta cell turnover observed in obese humans, they proposed that obesity-induced beta cell growth occurred by neogenesis through duct cell differentiation. However, the inherent limitations of post-mortem human samples prevent lineage tracing.

Various rodent models have demonstrated that adult beta cell growth and regeneration occur primarily by self-duplication [11–16]. However, metabolic demand is one of the most powerful stimuli for beta cell mass expansion [10, 17, 18] and thus obesity might alter beta cell fate through recruitment of specialised progenitors.

Several shortcomings limit current rodent models for the study of obesity-induced beta cell expansion. Long-term administration of a high-fat diet (HFD) can produce highly variable results [19] and beta cell expansion can take several weeks to occur [20]. Constitutive leptin receptor (LepR) signalling mutant models (*db/db* and *ob/ob* mice, *fa/fa* rats) develop obesity through germ line loss of leptin signalling. Reduced leptin signalling throughout embryonic development may provoke compensatory changes that limit the study of postnatal beta cells. These mutant rodents develop frank diabetes early in life, further complicating the study of beta cell turnover. An acute model of obesity is necessary to overcome these potential limitations. We developed a novel model of acute obesity to definitively clarify the lineage mechanism of beta cell mass expansion in obesity.

## Methods

### Mice

Experiments were performed at Baylor College of Medicine and Children’s Hospital of Philadelphia according to Institutional Animal Care and Use Committee protocols. *Rosa26*^(*CAG-Brainbow2.1*)^ (JAX no. 013731), *Rosa26*^(*loxP-stop-loxP-EYFP*)^ (JAX no. 006148) and *LepR*^*loxP/loxP*^ (JAX no. 008327) [21] mice were obtained from Jackson (Bar Harbor, ME, USA). *Ubc-Cre*^*ERT2*^ mice were from E. Brown at the University of Pennsylvania [22]. Crosses yielded *Ubc-Cre*^*ERT2*^*Rosa26*^*EYFP*^*LepR*^*loxP/loxP*^ and *Ubc-Cre*^*ERT2*^*Rosa26*^*(CAG-Brainbow2.1)*^*LepR*^*loxP/loxP*^ mice on a B6.129 F1 hybrid background, genotyped with REDExtract-N-Amp (Sigma-Aldrich, St Louis, MO, USA) (ESM Table 1). Male and female mice (5–6 weeks of age) were gavaged with tamoxifen (0.1 mg/g; MP, Santa Ana, CA, USA) for 5 days. Mice were labelled via drinking water with 5-bromo-2′-deoxyuridine (BrdU; 1 g/l; Sigma-Aldrich) or 5-ethynyl-2′-deoxyuridine (EdU; 0.5 g/l; Life Technologies, Grand Island, NY, USA), as described previously [23]. Intraperitoneal GTTs were performed as described previously [16]. Insulin tolerance tests (ITTs) were performed after 4 h fasting, using human regular insulin (1 U/kg; Eli Lilly, Indianapolis, IN, USA). Serum insulin was measured using a Mouse Ultrasensitive Insulin ELISA (Alpco Diagnostics, Salem, NH, USA). Mice were fed an HFD (60% of energy from fat; D12492; Research Diets, New Brunswick, NJ, USA) or chow diet (22% of energy from fat; No. 2919; Harlan, Houston, TX, USA). Randomisation of groups was not possible given the overt phenotype.

### Gene deletion

gDNA was extracted using Quick-gDNA MiniPrep (Zymo Research, Irvine, CA, USA). *LepR* gene deletion was assessed via Sybr Green (Sigma-Aldrich) qPCR (for primers see ESM Table 1).

### In vitro islet function

Islets isolated from individual mice at 1 week were cultured in RPMI 1640 medium with 10 mmol/l glucose and 10% fetal bovine serum for 2 days. Islet function was evaluated by perifusion as previously [24], with 3 mmol/l basal glucose (ramp of 0.625 mmol l^−1^ min^−1^), followed by 30 mmol/l KCl stimulation at completion. Insulin secretion was measured by HTRF assay (Cisbio, Bedford, MA, USA). Cytosolic calcium was measured as described previously [24]. Fura-2AM (Life Technologies) was used as a calcium indicator and was measured with a Zeiss AxioVision microscope (Carl Zeiss, Thornwood, NY, USA).

### Immunohistochemistry and morphometry

Paraffin sections were prepared as described previously [23]. Primary antisera included guinea pig anti-insulin (Dako, Carpinteria, CA, USA) and rat anti-BrdU (Accurate Chemical, Westbury, NY, USA), followed by secondary antisera conjugated to Cy2/Cy3 (Jackson ImmunoResearch, West Grove, PA, USA) and DAPI (Molecular Probes, Eugene, OR, USA). EdU was detected using Click-iT EdU Alexa Fluor 647 (Life Technologies) according to the manufacturer’s protocol. Slides were imaged to quantify beta cell morphometry as described previously [25], using Volocity 6.1.1 (PerkinElmer, Waltham, MA, USA). BrdU-positive, EdU-positive and BrdU/EdU co-positive beta cell ratios to total beta cells were calculated, and the percentage of predicted co-positive cells was obtained by dividing the percentage of actual co-positive cells by the percentage of predicted co-positive cells, multiplied by 100%. At least 3,000 beta cells were counted per mouse. Blinding of samples was not possible given the overt phenotype.

### Clonal analysis

Brainbow2.1 (*Rosa26*^*(CAG-Brainbow2.1)*^) expresses one of four colours (cyano-fluorescent protein [CFP], red fluorescent protein [RFP], yellow fluorescent protein [YFP] or green fluorescent protein [GFP]) from each allele [26, 27]. We were unable to reliably separate the spectral overlap for YFP and GFP, thus these were grouped together and represented as green in all images. Contiguous clones were identified as insulin-positive cells expressing a fluorescent protein(s) with a common border. Cells/clone were counted, with at least 230 clones counted per sample.

### Statistics

Results were reported as mean ± SEM unless noted otherwise, and compared with independent Student’s *t* tests (unpaired). No samples have been excluded from analysis.

## Results

### Acute *LepR* deficiency results in massive, progressive weight gain with moderate changes in glucose homeostasis

**Fig. 1:**
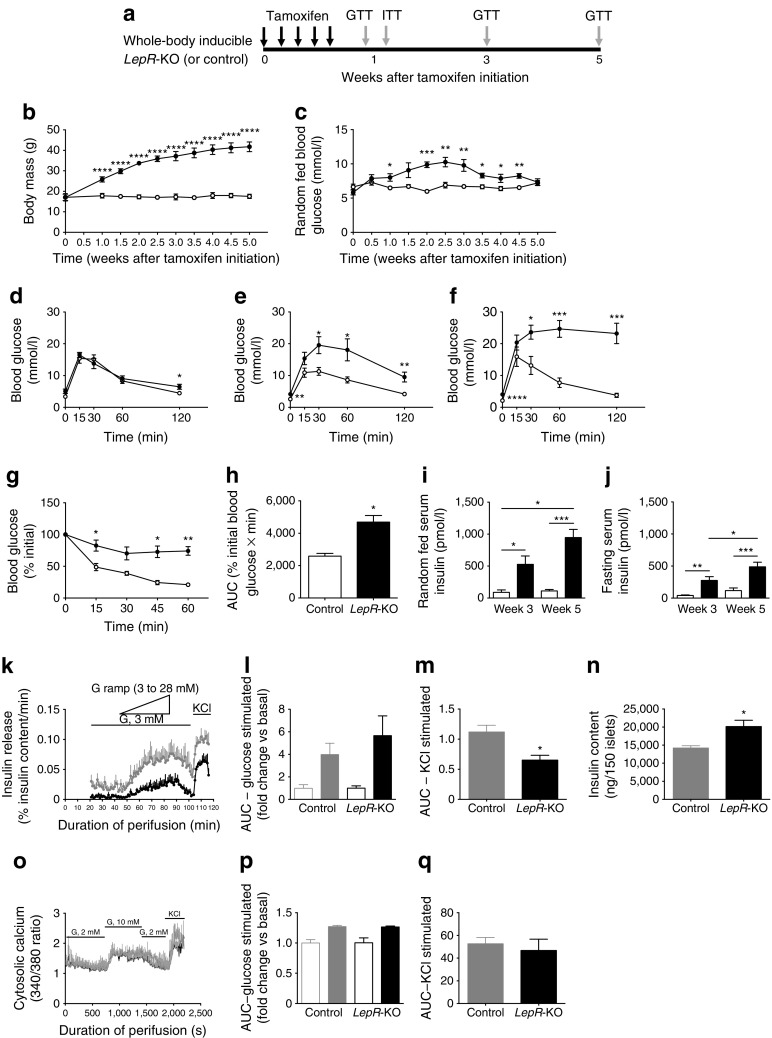
Acute *LepR* deficiency in mice results in massive, progressive weight gain with moderate changes in glucose homeostasis. (**a**) Timing of *LepR* gene deletion, washout, ITTs and GTTs. (**b**, **c**) Body mass (**b**) and randomly fed blood glucose levels (**c**) measured twice a week. (**d**–**f**) GTTs performed at day 5 (**d**), week 3 (**e**) and week 5 (**f**). (**g**, **h**) ITTs performed at week 1 (**g**); corresponding AUC is shown (**h**). (**i**, **j**) Random fed (**i**) and fasting (**j**) serum insulin. White circles and bars, control; black circles and bars, *LepR-*KO. Data are means ± SEM, except (**b**) (means ± SD); 3–7 mice per group. *p < 0.05, **p < 0.01, ***p < 0.001 and ****p < 0.0001 vs control. (**k**–**n**) Insulin release from isolated islets with glucose (G) ramp, followed by KCl stimulation (**k**), with corresponding AUC for glucose (normalised to baseline/group) (**l**) and KCl simulation (**m**), and total insulin content (**n**). (**o**–**q**) Cytosolic calcium release from isolated islets (**o**) and corresponding AUC for glucose (normalised to baseline/group) (**p**) and KCl stimulation (**q**). Grey traces, control; black traces, *LepR-*KO; white bars, basal; black/grey bars, stimulated. Data are means ± SEM, 3 mice per group. *p < 0.05 vs control

We created a unique model of obesity by deriving whole-body *LepR*-deficient (*Ubc-Cre*^*ERT2*^*LepR*^*loxP/loxP*^; *LepR-*knockout [KO]) mice on a B6.129 F1 hybrid background, resulting in tamoxifen-induced *LepR* gene deletion (ESM Fig. 1). We characterised the metabolic phenotype of *LepR*-KO and *LepR*^*loxP/loxP*^ littermate controls (Fig. 1a). Both sexes demonstrated equivalent phenotypical characteristics following *LepR* deletion (ESM Tables 2, 3). *LepR*-KO mice displayed extreme weight gain, with their weight having increased by more than twofold by week 5 (Fig. 1b; control 17.8 g vs *LepR*-KO 41.7 g; *p* < 0.0001). Random fed blood glucose levels transiently increased in *LepR*-KO mice, returning to control values by 5 weeks (Fig. 1c). Glucose tolerance gradually declined in *LepR-*KO mice, with significantly elevated fasting blood glucose concentrations (Fig. 1d–f). Insulin resistance developed in *LepR*-KO mice within 1 week (Fig. 1g, h). Thus, acute *LepR* deletion leads to extreme obesity with moderate changes in glucose homeostasis.

*LepR-*KO mice do not develop frank diabetes by compensating, in part, through increased beta cell function. Fed and fasting serum insulin levels were increased in *LepR-*KO mice compared with controls (Fig. 1i, j); measurement of basal and glucose-stimulated insulin secretion suggested that *LepR*-KO islets secreted less insulin despite a 41% increase in total insulin content compared with controls (Fig. 1k–n). Glucose stimulation index was preserved in *LepR-*KO islets (Fig. 1l), as was stimulated calcium release (Fig. 1o–q). Importantly, *LepR*-KO islets remain responsive to glucose, despite blunted basal and stimulated insulin release. Thus, islet dysfunction did not confound our ability to analyse beta cell mass expansion in response to acute obesity.

### Acute *LepR* deficiency results in massive, rapid beta cell mass expansion

**Fig. 2:**
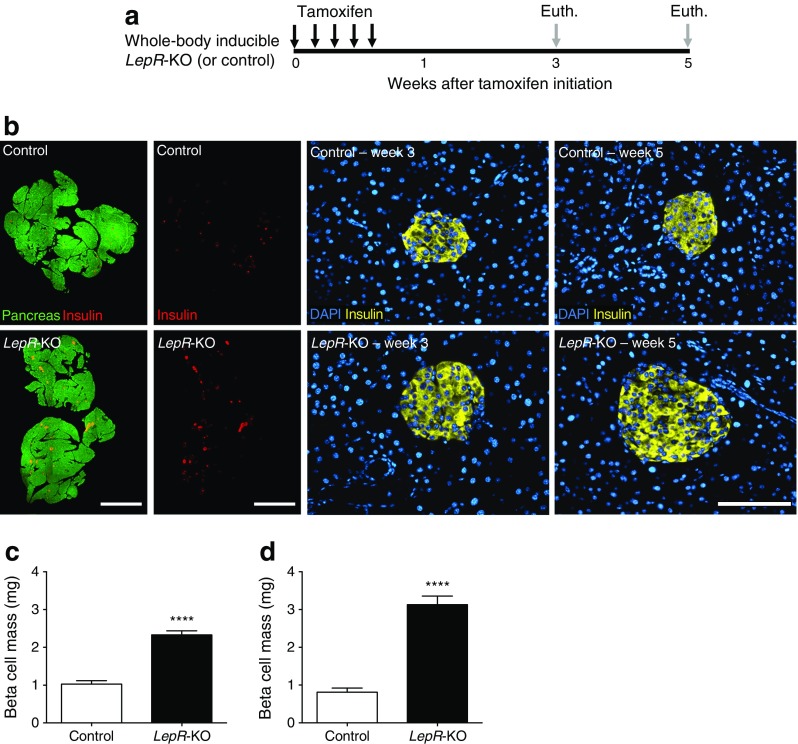
Acute *LepR* deficiency results in massive, rapid beta cell mass expansion. (**a**) Timing of *LepR* gene deletion and euthanasia (Euth). (**b**) Total pancreas (green) and total beta cell area (red), generated by composites of low-power image scans, and high-power images of insulin-positive beta cells (yellow; DAPI in blue) for control and *LepR*-KO mice. Scale bars, 2 mm low power and 100 μm high power. (**c**, **d**) Beta cell mass at 3 weeks (**c**) and 5 weeks (**d**) in control (white bars) and *LepR*-KO (black bars) mice. Data are means ± SEM, 6–10 sections per pancreas, 5–7 mice per group. ****p < 0.0001 vs control

We hypothesised that beta cells expand in response to extreme obesity to meet the increasing insulin demand. We measured beta cell morphometry at various times after tamoxifen initiation (Fig. 2a). Beta cell mass increased by more than twofold in *LepR-*KO mice compared with control mice at 3 weeks and was further increased at 5 weeks (Fig. 2b–d and ESM Table 4). Beta cell size and cross-sectional beta cell area per islet were greater in *LepR-*KO mice and there were more large islets (ESM Table 5). Thus, obesity associated with acute *LepR* deficiency induced massive, rapid beta cell expansion.

### Acute *LepR* deficiency rapidly and massively increases beta cell proliferation

**Fig. 3:**
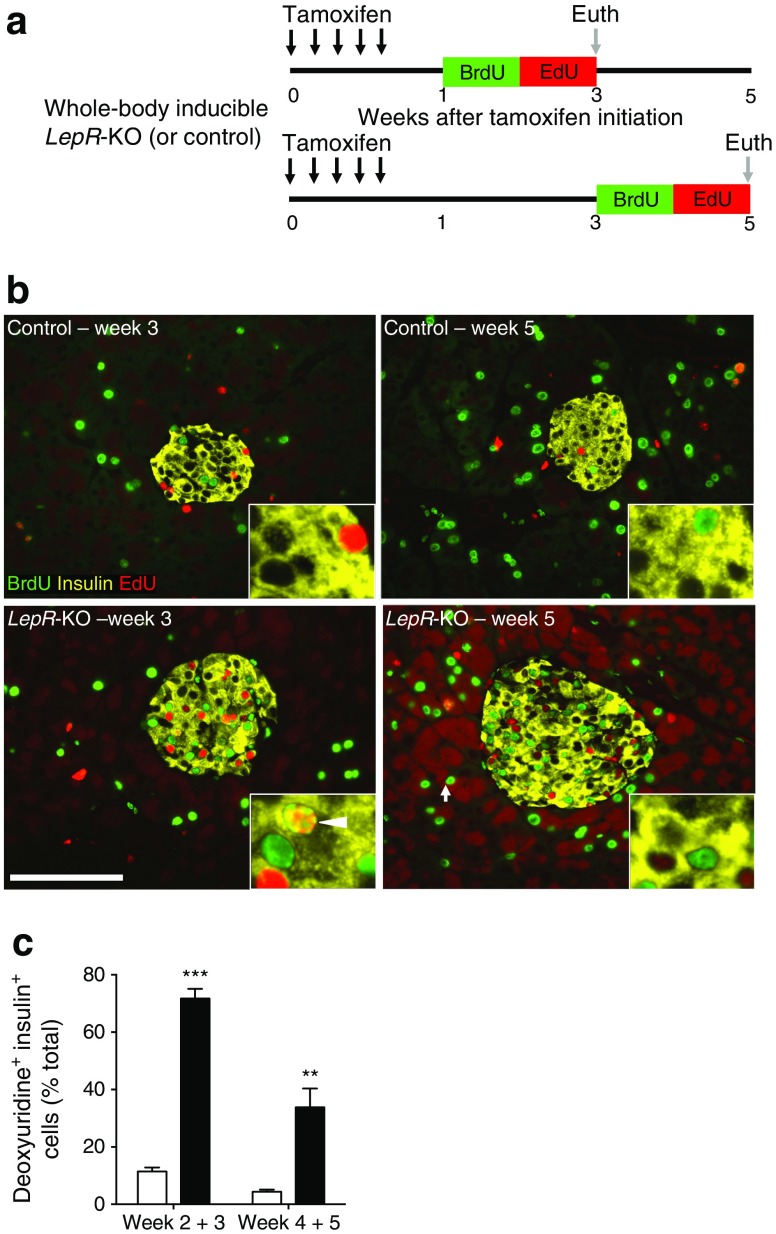
Acute *LepR* deficiency rapidly and massively increases beta cell proliferation. (**a**) Timing of *LepR* gene deletion, thymidine analogue labelling, and euthanasia (Euth). (**b**) BrdU (green), insulin (yellow) and EdU (red) detection for control and *LepR*-KO islets. Arrowhead indicates BrdU/EdU co-positive (BrdU^+^ EdU^+^) beta cell. Scale bar, 100 μm. (**c**) Cumulative beta cell proliferation, measured by deoxyuridine-positive (BrdU and EdU) insulin-positive cells as a percentage of total beta cells, from control (white bars) and *LepR*-KO (black bars) mice. Data are means ± SEM from ≥3,000 beta cells per pancreas, 6–9 mice per group. **p < 0.01, ***p < 0.001 vs control

To examine the impact of acute obesity upon beta cell proliferation, we administered thymidine analogues for 2 weeks before mice were euthanised (Fig. 3a). Two-week thymidine incorporation in beta cells of control mice was 11% at 3 weeks and declined to 5% at 5 weeks (Fig. 3b, c and ESM Table 6). Beta cell proliferation dramatically increased to 70% in *LepR-*KO mice at 3 weeks. Despite continued metabolic demand at 5 weeks (Fig. 1), beta cell proliferation declined to 34% (Fig. 3b, c and ESM Table 6). Notably, acute *LepR* deficiency represents one of the strongest known stimuli for beta cell replication.

### Beta cells expand by self-renewal in acute *LepR*-deficient mice

**Fig. 4:**
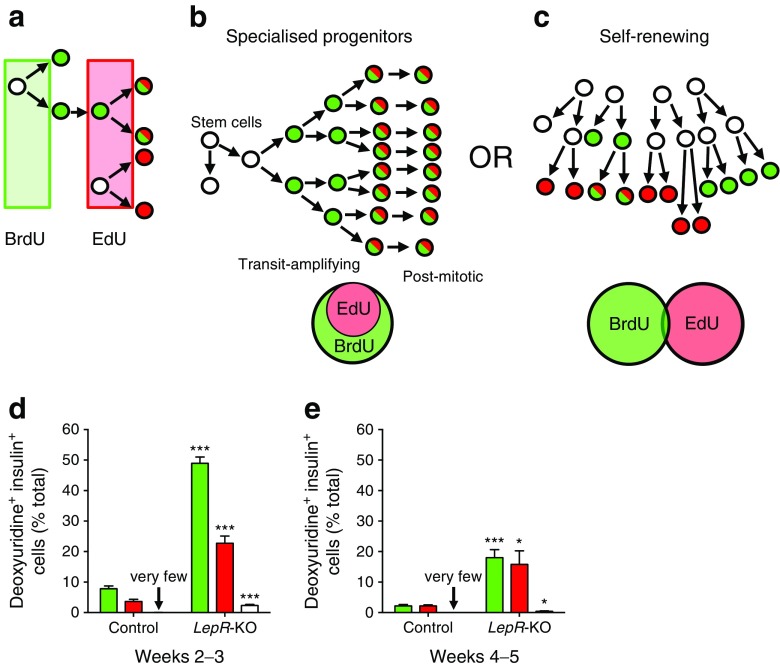
Beta cells expand by self-renewal in acute *LepR*-deficient mice. (**a**) Labelling sequential cell divisions with BrdU (green) and EdU (red) results in co-labelled cells (green/red; reproduced from [14] with the permission of Elsevier). (**b**, **c**) Potential mechanisms of cell expansion employ specialised progenitors or self-renewing cell division (reproduced from [14] with permission of Elsevier). Specialised progenitor lineages exhibit sequential cell division (**b**), resulting in BrdU/EdU co-labelled cells. Self-renewing lineages exhibit random cell division (**c**), with few co-labelled cells. (**d**, **e**) Total beta cell accumulation of BrdU (green), EdU (red) or both (white) for weeks 2–3 (**d**) and 4–5 (**e**). Data are means ± SEM, ≥3,000 beta cells per pancreas, 6–9 mice per group. *p < 0.05 and ***p < 0.001 vs control

We tested if beta cells expand in obesity via highly proliferative ‘transit amplifying’ progenitors, as observed in skin or intestine. To determine the lineage mechanism of beta cell expansion, we employed sequential administration of two thymidine analogues (Fig.4a), as described previously [14, 16]. By labelling the first cell division with BrdU (green) and the second with EdU (red), sequential cell division resulted in BrdU/EdU co-labelled cells (Fig. 3b inset, Fig. 4a–c, [14]). If beta cells expand by specialised progenitors undergoing sequential cell division (transit amplifying population), then the beta cells would be BrdU/EdU double-positive (Fig. 4b). Alternatively, if beta cells expand by self-renewal through random cell division, few double-positive beta cells would be expected after obesity (Fig. 4c). During week 2 in control mice, 8% of beta cells incorporated BrdU while during week 3, ∼4% of beta cells were labelled with EdU (Fig. 4d). BrdU/EdU co-positive beta cells were nearly absent in control mice (Fig. 4d, e and ESM Table 6), consistent with previous reports of beta cell expansion [14]. In contrast, acute *LepR* deletion resulted in labelling of ∼50% of beta cells in week 2 and 23% in week 3. Despite substantial beta cell proliferation in *LepR-*KO mice, only a tiny fraction of beta cells were BrdU/EdU co-positive (2.3%). Beta cell proliferation declined during weeks 4 and 5 in *LepR-*KO mice, but remained significantly elevated compared with controls, at 15–18% (Fig. 4e). Accordingly, even fewer BrdU/EdU co-positive beta cells were observed relative to weeks 2–3. Thus, during 2 weeks of immense beta cell proliferation following *LepR* deficiency, beta cells usually divided once and rarely divided twice. This strongly supports a lineage mechanism of self-renewal (Fig. 4c) as the primary source of new beta cells.

### Acute *LepR* deficiency does not bypass the replication refractory period of beta cell turnover

**Fig. 5:**
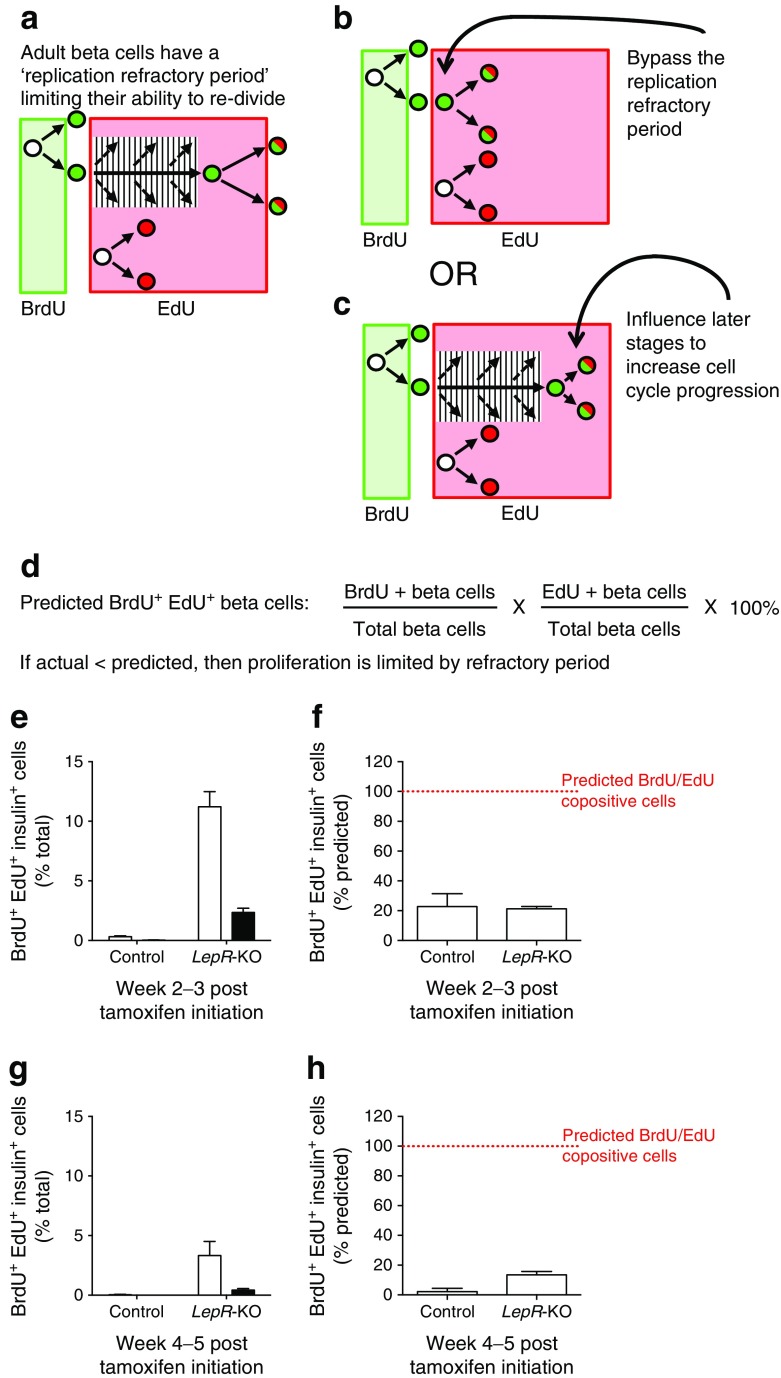
Acute *LepR* deficiency does not bypass the replication refractory period of beta cell turnover. (**a**) Adult beta cells are post-mitotic but can eventually divide more than once, limited by the replication refractory period [14]. (**b**, **c**) Opposing models of beta cell expansion suggest that increased beta cell replication results from bypassing the replication refractory period (**b**) or increasing cell cycle progression at later cell cycle stages (**c**). (**d**) Equation for predicted BrdU/EdU co-positive cells. (**e**–**h**) Quantification of BrdU/EdU co-positive beta cells for the predicted (white) and actual (black) co-positive beta cells (% total beta cells) (**e**, **g**) and the actual BrdU/EdU co-positive beta cells expressed as a percentage of predicted BrdU/EdU co-positive beta cells (red line) (**f**, **h**). Data are means ± SD, ≥3,000 beta cells counted per pancreas, 6–9 mice per group

Adult beta cell replication is governed by a ‘replication refractory period’, which limits beta cell turnover from occurring a second time in a recently divided beta cell (Fig. 5a) [14, 28]. However, the replication refractory period of beta cells does not appear to be permanently set, but might be altered under some conditions [14, 28]. Acute *LepR* deficiency-induced obesity is one of the strongest stimuli for beta cell replication, and therefore might bypass the replication refractory period (Fig. 5b). Alternatively, obesity-induced beta cell expansion could influence cell cycle progression at a later stage, without altering the refractory period (Fig. 5c). If beta cell replication is stochastic, the proportion of BrdU/EdU co-positive beta cells should be equal to the predicted fraction of BrdU/EdU co-positive beta cells (Fig. 5d) [14]. Alternatively, if beta cell replication is not stochastic, BrdU/EdU co-positive beta cells would be less frequent than predicted (Fig. 5d). At 3 weeks very few BrdU/EdU co-positive beta cells were observed in control mice (Fig. 5e and ESM Table 6). BrdU/EdU co-positive cells in controls represented ∼23% of the predicted co-positive population, substantially less than 100% (stochastic cell division) (Fig. 5f), indicating that the beta cell replication refractory period is not foreshortened [14]. BrdU/EdU co-positive beta cells were more frequent in *LepR-*KO mice (2.3%), but remained considerably less than the predicted value (21%; Fig. 5e, f). This observation of extremely low numbers of double-dividing cells relative to numbers predicted persisted during weeks 4–5 (Fig. 5g, h). These results suggest that acute *LepR* deficiency does not shorten the beta cell replication refractory period to less than the 2 week labelling period. Therefore, the signals associated with obesity-induced beta cell expansion likely act to stimulate a pool of beta cells ‘licensed’ to proliferate rather than recruiting recently divided beta cells to divide again (Fig. 5c).

### Beta cells do not clonally expand in acute *LepR* deficiency

**Fig. 6:**
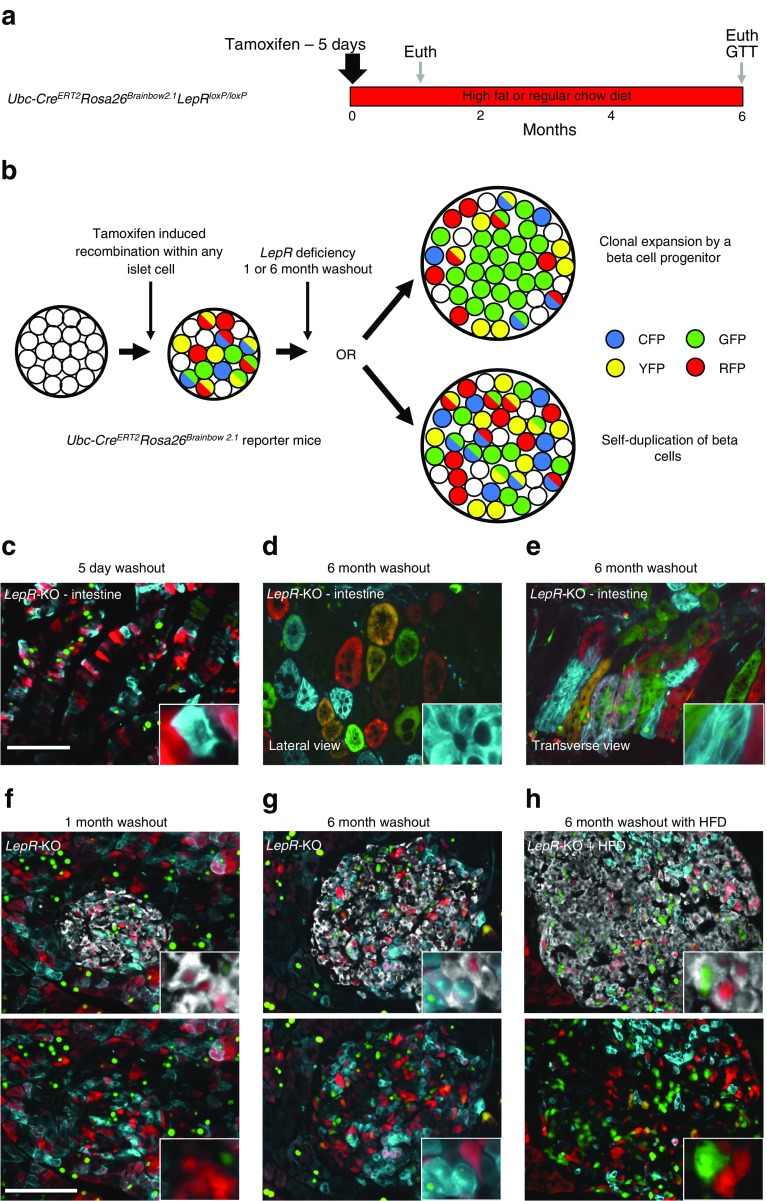
Beta cells do not clonally expand in acute *LepR* deficiency. (**a**) Timing of tamoxifen-induced recombination in *Ubc-Cre* ^*ERT2*^ *Rosa26* ^*(CAG-Brainbow2.1)*^ *LepR* ^*loxP/loxP*^ mice. Cell labelling by Brainbow2.1 [26] was followed by 1 or 6 month washout on regular diet or HFD, and euthanasia (Euth). (**b**) Opposing models of beta cell expansion using the *Ubc-Cre* ^*ERT2*^ *Rosa26* ^*(CAG-Brainbow2.1)*^ reporter: islets comprised of a large monochromatic clone indicate cells of common origin via a rapidly dividing progenitor; islets featuring single or double cell clones suggest expansion occurs through self-duplication. (**c**–**e**) Intestine from *Ubc-Cre* ^*ERT2*^ *Rosa26* ^*(CAG-Brainbow2.1)*^ *LepR* ^*loxP/loxP*^ mice after 5 days (**c**) and 6 months (**d**, **e**), demonstrating clonal expansion in lateral (**d**) and transverse (**e**) views. (**f**–**h**) Images following 1 month (**f**) or 6 months (**g**) on a regular diet or 6 months on an HFD (**h**)

To further test for specialised progenitor cells in obesity, we used *Cre* loxP-based clonal analysis of beta cells. In the Brainbow2.1 model, tamoxifen induces random expression of one of four fluorescent proteins (CFP, RFP, GFP or YFP) from each allele. We crossed *Ubc-Cre*^*ERT2*^*LepR*^*loxP/loxP*^ with confetti (*Rosa*^*(CAG-Brainbow2.1)*^) to generate homozygous *LepR*-KO confetti mice (*Ubc-Cre*^*ERT2*^*Rosa*^*(CAG-Brainbow2.1*^*LepR*^*loxP/loxP)*^), which were treated with tamoxifen and then euthanised at various times (Fig. 6a, b and ESM Table 7). GFP and YFP were considered one distinct population represented in green (due to spectral overlap), resulting in six potential outcomes for cell labelling (CFP, RFP, GFP/YFP, CFP+RFP, CFP+GFP/YFP and RFP+GFP/YFP) that provided high-level resolution of clones within the islet.

We hypothesised that clonal expansion by a beta cell progenitor would lead to a single dominant monochromatic clone within islets (Fig. 6b). Alternatively, beta cell expansion by self-duplication would produce islets comprised of multiple small clones of different colours. Intestinal crypts were initially multicoloured but demonstrated clonal expansion after 6 months giving rise to monochromatic villi (Fig. 6c–e) [27], indicating the contribution of a stem cell population to intestinal epithelia, as previously reported [27]. In contrast, pancreatic islets did not demonstrate clonal expansion, even after a 6 month washout (Fig. 6f, g and ESM Fig. 2). Labelled cells within islets were mainly found in isolation, with a few contiguous clones of between two and seven cells observed at lower frequencies than single cell clones (ESM Table 8). We then attempted to push the limits of beta cell expansion by placing *LepR*-KO mice on an HFD to maximise obesity-induced recruitment of new beta cells (Fig. 6h and ESM Fig. 2). These mice exhibited massive weight gain (+41.3 g). However, even HFD-fed *LepR-*KO mice did not exhibit expansion of a single dominant beta cell clone in any islet. We found no evidence of islet neogenesis in any cohort examined, as all islets surveyed were multicoloured (ESM Table 8). We conclude that acute *LepR* deficiency does not induce clonal expansion from a beta cell progenitor.

## Discussion

Using a novel model of inducible obesity and state-of-the-art lineage tracing tools, we find that extreme metabolic demand does not alter beta cell fate. Previous attempts to induce acute *LepR* deletion using *Rosa-Cre*^*ERT2*^;*LepR*^*flox/flox*^ mice did not result in obesity due to inadequate floxing of *LepR* in the brain [29]. In contrast, *LepR-*KO mice, via *Ubc-Cre*^*ERT2*^*LepR*^*loxP/loxP*^, doubled their body mass in 3 weeks. *LepR*-KO mice developed impaired glucose tolerance and insulin resistance in the absence of frank diabetes, with restoration of blood glucose through increased beta cell function and mass. Beta cell mass expansion resulted from significant beta cell proliferation without the contribution of a highly replicative progenitor cell or clonally expanding beta cells. Additionally, BrdU/EdU co-positive cells were observed in lower frequencies than predicted by chance, indicating that even during increased metabolic demand, beta cells are still restricted by the replication refractory period.

Acute *LepR* deficiency dramatically increased beta cell mass in response to obesity, by 3.7-fold at 5 weeks. In comparison, HFD feeding in young mice required several months to reach the magnitude of beta cell mass expansion observed in our model [19, 30, 31]. Constitutive leptin- or *LepR*-deficient mice (*ob/ob* and *db/db*) have equivalent beta cell mass to our inducible *LepR-*KO mice [17, 18, 32, 33]. These observations reinforce the impact of our inducible model to expand beta cells, with the ability to specifically test postnatal beta cell expansion without development of frank diabetes. Euglycaemia was maintained in *LepR-*KO mice for at least 6 months (ESM Table 7). Importantly, nondiabetic obese people exhibit compensatory beta cell expansion [4, 10]. Therefore, inducible obese mice represent a compelling model with which to interrogate the signals governing mammalian beta cell expansion.

Acute *LepR* deficiency stimulated massive beta cell proliferation, which was greatest in weeks 2 and 3, with thymidine incorporation rates of 50% (7.1% per day) and 23% (3.3% per day), respectively. These observations far exceed previous results employing mitogenic stimuli such as partial pancreatectomy (PPx), streptozotocin, exendin-4, pregnancy, HFD and glucose [14, 28, 34–36]. Adult beta cell replication is strictly regulated by a replication refractory period that limits beta cell turnover from one round of the cell cycle to the next [14, 28]. In our previous models (PPx, exendin-4, pregnancy) very few beta cells incorporated dual thymidine labels over short periods, suggesting that the refractory period is absolute and cannot be overcome by regenerative stimuli [14]. However, these studies did not include obesity, one of the most powerful stimuli for beta cell expansion. Thus, we were unable to address whether the beta cell replication refractory period is absolute or relative. Therefore, we employed the strongest tool for beta cell proliferation, using acute massive obesity to definitively test the limits of the beta cell replication refractory period. The remarkable magnitude and rapid occurrence of beta cell proliferation in *LepR*-KO mice was far greater than observed in previous models, hinting that obesity-induced beta cell proliferation might exceed the limitations of the refractory period. But, despite highly elevated beta cell proliferation in *LepR*-KO mice, acute inducible obesity did not shorten the beta cell replication refractory period. This sharply contrasts with other studies on glucose and connective tissue growth factor, which advance potential beta cell mitogens capable of shortening the beta cell replication refractory period [28, 37]. Although *LepR-*KO mice were hyperglycaemic during thymidine labelling, the length of the refractory period did not decrease. A greater understanding of the mechanisms that govern the beta cell replication refractory period is important for identifying potential therapeutic targets to expand beta cells for patients with diabetes.

Acute *LepR* deficiency adds to the weight of evidence that beta cells expand by self-renewal. Previous studies revealed that adult beta cell regeneration largely occurs by self-duplication of pre-existing beta cells [11, 14–16]. Extensive studies have attempted to identify a stimulus that might recruit stem/progenitor cell differentiation in beta cell regeneration. Metabolic demand potently expands beta cell mass but the lineage mechanism of beta cell growth in obesity remained unknown. Researchers have speculated that stem cells potentially contribute to beta cell expansion in obesity [10, 38]. Islet ‘stem cells’ would presumably involve a transit amplifying population and undergo multiple rounds of cell division incorporating both BrdU and EdU in beta cells. However, in our studies such double-labelled BrdU/EdU insulin-positive beta cells were very rare, even in response to massive obesity-associated beta cell expansion. Although our results from short-term thymidine analogue labelling do not completely rule out contribution by a specialised progenitor to beta cell expansion, this seems highly unlikely. Taken together, our results further support the hypothesis that self-renewal of beta cells is the primary mechanism of beta cell expansion.

Elegant tools have been developed to study the clonal origins of mature differentiated cells. Brainbow2.1 has been used to examine clonal expansion of intestinal stem cells [27, 39], neuronal fate [26, 40], salivary glands [41] and cardiac growth [42]. We employed Brainbow2.1 for clonal analysis of beta cell expansion in obesity. We did not find any monochromatic islets in *LepR*-deficient mice, indicating that islets do not arise entirely from a common single cell at the time of labelling. Furthermore, we did not detect the presence of a single dominant clone within any islets. In contrast, we frequently found multiple small clones within individual islets. We found similar trends in *LepR-*KO mice challenged with an HFD. Previous reports have used low-frequency labelling of beta cells with the mosaic analysis with double markers (MADM) reporter to examine beta cell fate within rare cells [43, 44]. These studies demonstrated that beta cell clones were small and of similar size in normal adult beta cell growth. Our studies extend this work, showing that obesity-induced beta cell growth does not occur through clonal expansion.

The signalling mechanism(s) of beta cell expansion remain uncertain, although recent studies suggest that endoplasmic reticulum (ER) stress may regulate beta cell proliferation [45, 46]. The increased insulin demand and subsequent hyperinsulinaemia following acute *LepR* deletion may stimulate ER-stress-induced beta cell proliferation [45]. Further investigation is necessary to discern the relationship between insulin demand, ER-stress and beta cell proliferation in *LepR*-KO mice.

In summary, we developed a unique model of inducible obesity with which to determine the developmental mechanisms of obesity-associated beta cell mass expansion. Acute *LepR* deficiency represents the strongest stimulus for studying the lineage mechanisms of beta cell growth, but no evidence of a highly replicative progenitor cell or clonal expansion was found. Efforts to expand beta cells should continue to focus on identifying the signals that stimulate and regulate cell cycle progression. Moreover, acute *LepR* deficiency is an excellent model for future studies to interrogate the signals regulating beta cell growth for the development of potential diabetes therapies.

## Electronic supplementary material

Below is the link to the electronic supplementary material.

ESM Fig. 1 (PDF 7 kb)

ESM Fig. 2 (PDF 549 kb)

ESM Table 1 (PDF 40 kb)

ESM Table 2 (PDF 60 kb)

ESM Table 3 (PDF 41 kb)

ESM Table 4 (PDF 69 kb)

ESM Table 5 (PDF 64 kb)

ESM Table 6 (PDF 81 kb)

ESM Table 7 (PDF 55 kb)

ESM Table 8 (PDF 49 kb)

## Abbreviations

BrdU: 5-Bromo-2′-deoxyuridine
CFP: Cyano-fluorescent protein
EdU: 5-Ethynyl-2′-deoxyuridine
ER: Endoplasmic reticulum
GFP: Green fluorescent protein
HFD: High-fat diet
ITT: Insulin tolerance test
KO: Knockout
LepR: Leptin receptor
PPx: Partial pancreatectomy
RFP: Red fluorescent protein
YFP: Yellow fluorescent protein
