# Supplementary material for: Extreme obesity induces massive beta cell expansion in mice through self-renewal and does not alter the beta cell lineage
Source: Diabetologia. 2016 Mar 22;59:1231–41. doi: 10.1007/s00125-016-3922-7 (PMC4869735; doi:10.1007/s00125-016-3922-7)
Supplement: Supplementary file 1 — (PDF 7 kb) [file 125_2016_3922_MOESM1_ESM.pdf]

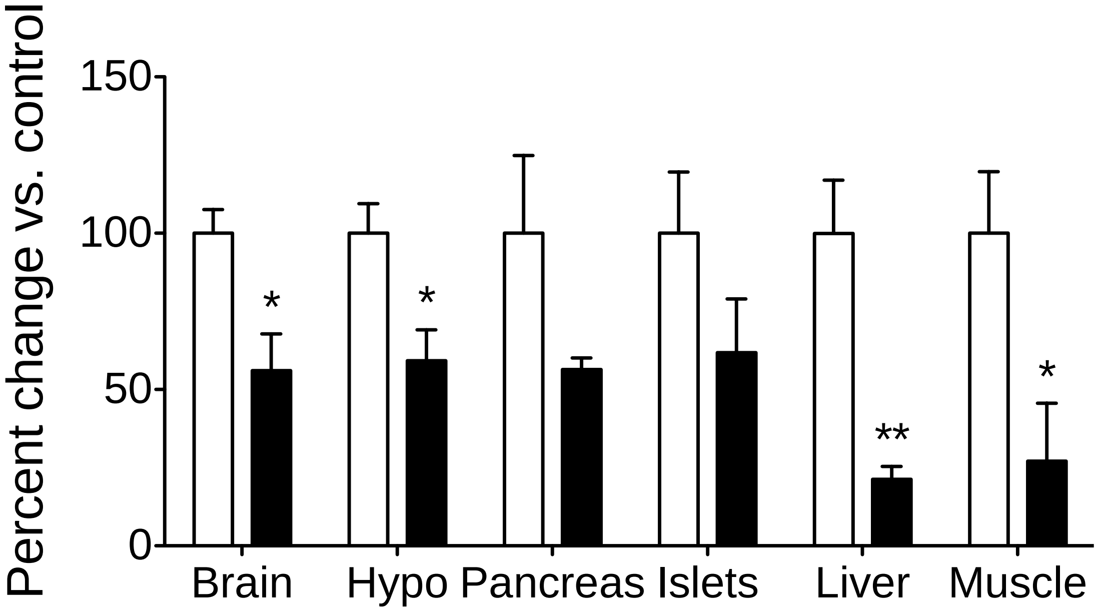

ESM Fig. 1. The leptin receptor gene (*LepR*) is significantly reduced in tamoxifen-treated *Ubc-Cre*^{ERT2} *LepR*$^{loxP/loxP}$ mice. Quantitative PCR analysis from brain, hypothalamus, pancreas, isolated islets, liver, and muscle in *LepR*$^{loxP/loxP}$ (control; white bars) and *Ubc-Cre*^{ERT2} *LepR*$^{loxP/loxP}$ (black bars) mice represented as the percentage change versus control for each tissue. Mean±SEM, 3-4 animals/group. *$p<0.05$, **$p<0.01$ versus control.
