# Supplementary material for: Extreme obesity induces massive beta cell expansion in mice through self-renewal and does not alter the beta cell lineage
Source: Diabetologia. 2016 Mar 22;59:1231–41. doi: 10.1007/s00125-016-3922-7 (PMC4869735; doi:10.1007/s00125-016-3922-7)
Supplement: Supplementary file 2 — (PDF 549 kb) [file 125_2016_3922_MOESM2_ESM.pdf]

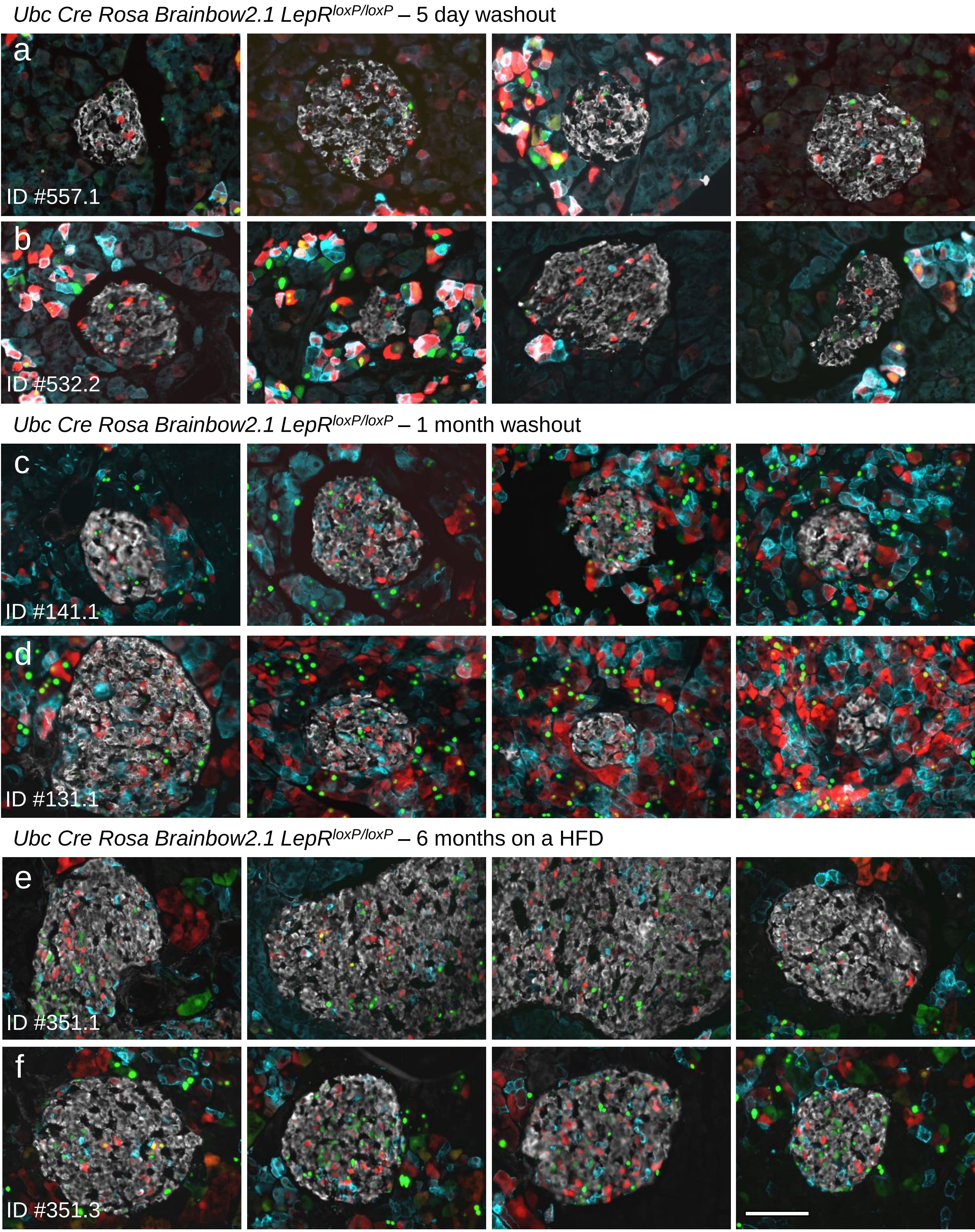

Cox et al ESM. Fig. 2

ESM Fig. 2. Representative images of Brainbow2.1 reporter labelling beta cells at various time points. Tamoxifen-induced recombination in *Ubc-Cre*^{ERT2} *Rosa26*$^{(CAG\text{-}Brainbow2.1)}$ *LepR*$^{loxP/loxP}$ mice was performed and pancreata were harvested (a-b) on day 5, the last day of tamoxifen treatment, after (c-d) 1 month washout, and (e-f) 6 months washout on a high fat diet (HFD). Four representative images are shown per mouse, from two mice per cohort with their respective animal ID # indicated.
