# Supplementary material for: Extreme obesity induces massive beta cell expansion in mice through self-renewal and does not alter the beta cell lineage
Source: Diabetologia. 2016 Mar 22;59:1231–41. doi: 10.1007/s00125-016-3922-7 (PMC4869735; doi:10.1007/s00125-016-3922-7)
Supplement: Supplementary file 3 — (PDF 40 kb) [file 125_2016_3922_MOESM3_ESM.pdf]

ESM Table 1. Genoptyping and qPCR primers (5' - 3'). Ubc Cre, LepR loxP/loxP, Rosa26 YFP, and Rosa26 confetti primers were used to genotype mice. InsR primers were used as a control gene for qPCR with LepR loxP/loxP primers

Gene	Forward	Reverse	
**Ubc Cre**	CGCTCGGGGTTGGCGAGTGTGTTTTGTGAA	GCCTGGCGATCCCTGAACATGTCCATCAGG	
**LepR loxP/loxP**	GTTGTTTGTAAGCTACAAGGCTGTATG	GTACAGGTCTCTTTGCTAAACCTGAAG	
**InsR**	TCCACTTTAGAGTAACAGACTGAGGTT	AAGTTAAGAGGGGAATGTGTGGAATAC	
**Gene**	**Common**	**Wild Type**	**Mutant**
**Rosa26 YFP**	AAAGTCGCTCTGAGTTGTTAT	GGAGCGGGAGAAATGGATATG	AAGACCGCGAAGAGTTTGTC
**Rosa26 Confetti**	CCAGATGACTACCTATCCTC	AAAGTCGCTCTGAGTTGTTAT	GAATTAATTCCGGTATAACTTCG
