# Supplementary material for: Extreme obesity induces massive beta cell expansion in mice through self-renewal and does not alter the beta cell lineage
Source: Diabetologia. 2016 Mar 22;59:1231–41. doi: 10.1007/s00125-016-3922-7 (PMC4869735; doi:10.1007/s00125-016-3922-7)
Supplement: Supplementary file 4 — (PDF 60 kb) [file 125_2016_3922_MOESM4_ESM.pdf]

**ESM Table 2. Individual physiologic data from LepR lp/lp and Ubc Cre LepR lp/lp mice.** Initial and final body weight (g), initial and final blood glucose (mmol/l), glucose tolerance test blood glucose measurements (mmol/l) after overnight fast (mmol/l) and at time 15, 30, 60, and 120 min followed by the area under the curve (AUC; mmol/l x min), random fed serum insulin (ng/ml), insulin tolerance test blood glucose measurements (mmol/l) after a 4 hour fast and at time 0, 15, 30, 45, and 60 min followed by the area under the curve (AUC; % initial blood glucose x min), gonadal fat weight (g). Measurements were made in three different cohorts of LepR lp/lp and Ubc Cre LepR lp/lp mice at 3 and 5 weeks after tamoxifen initiation.

**Week 3**

| | Group | ID Number | Sex | # | Initial Wt (g) | Final Wt (g) | Init. BG (mmol/l) | Final BG (mmol/l) | Fast BG (mmol/l) | 15 min BG (mmol/l) | 30 min BG (mmol/l) | 60 min BG (mmol/l) | 120 min BG (mmol/l) | AUC (mmol/l x min) | Fed Insulin (pmol/l) | Fasting Insulin (pmol/l) | 4 hr Fast BG (mmol/l) | 0 min BG (mmol/l) | 15 min BG (mmol/l) | 30 min BG (mmol/l) | 45 min BG (mmol/l) | 60 min BG (mmol/l) | AUC (% initial x min) | Gonad Fat (g) |
|---|---|---|---|---|---|---|---|---|---|---|---|---|---|---|---|---|---|---|---|---|---|---|---|---|
| Control | LepR lp/lp | 62.3 | M | 1 | 21.2 | 21.4 | 8.4 | 6.0 | 3.3 | 12.6 | 11.8 | 9.7 | 5.3 | 1076 | 62.5 | 44.4 | ND | ND | ND | ND | ND | ND | ND | ND |
| | LepR lp/lp | 63.2 | F | 2 | 16.9 | 18.2 | 7.5 | 6.6 | 2.7 | 12.7 | 13.6 | 10.6 | 3.6 | 1100 | ND | 73.2 | ND | ND | ND | ND | ND | ND | ND | ND |
| | LepR lp/lp | 63.3 | F | 3 | 19.3 | 19.2 | 7.7 | 6.9 | 2.7 | 10.6 | 11.3 | 9.0 | 4.1 | 960 | 39.3 | 54.4 | ND | ND | ND | ND | ND | ND | ND | ND |
| | LepR lp/lp | 63.4 | F | 4 | 18.4 | 18.3 | 6.9 | 6.9 | 2.4 | 13.2 | 13.2 | 8.9 | 4.2 | 1038 | 56.8 | 22.0 | ND | ND | ND | ND | ND | ND | ND | ND |
| | LepR lp/lp | 64.3 | F | 5 | 16.8 | 15.2 | 6.1 | 5.1 | 1.8 | 5.4 | 6.7 | 4.9 | 3.8 | 581 | ND | 58.7 | ND | ND | ND | ND | ND | ND | ND | ND |
| | LepR lp/lp | 22.2Bl | M | 6 | 22.6 | 23.6 | 10.5 | 8.6 | 4.1 | 6.0 | 6.2 | 6.3 | 4.5 | 678 | 318.9 | ND | 9.4 | 10.0 | 5.2 | 3.4 | 2.0 | 1.8 | 4448 | 279 |
| | LepR lp/lp | 24.4Bl | M | 7 | 20.7 | 21.0 | 11.9 | 10.1 | 3.4 | 22.5 | 21.3 | 11.6 | 5.1 | 1516 | 101.4 | 50.4 | 10.4 | 10.8 | 6.1 | 4.8 | 3.3 | 2.7 | 5663 | 190 |
| | LepR lp/lp | 25.1Bl | M | 8 | 19.4 | 20.5 | 11.3 | 9.2 | 6.3 | 27.7 | 22.1 | 13.1 | 7.2 | 1765 | 136.4 | 24.3 | 9.1 | 12.0 | 4.6 | 4.6 | 2.7 | 2.3 | 5130 | 92 |
| | Average | | | | 19.4 | 19.7 | 8.8 | 7.4 | 3.3 | 13.8 | 13.3 | 9.3 | 4.7 | 1089.3 | 119.2 | 46.8 | 9.6 | 10.9 | 5.3 | 4.3 | 2.7 | 2.3 | 5080.3 | 187.0 |
| | SEM | | | | 0.7 | 0.9 | 0.8 | 0.6 | 0.5 | 2.7 | 2.1 | 0.9 | 0.4 | 139.2 | 42.4 | 7.0 | 0.4 | 0.6 | 0.5 | 0.4 | 0.4 | 0.3 | 351.6 | 54.0 |
| LepR KO | Ubc Cre LepR lp/lp | 62.1 | M | 1 | 20.5 | 33.7 | 10.9 | 9.8 | 4.9 | 20.7 | 22.4 | 13.3 | 5.9 | 1625 | 291.0 | 91.6 | ND | ND | ND | ND | ND | ND | ND | ND |
| | Ubc Cre LepR lp/lp | 62.2 | M | 2 | 20.8 | 27.1 | 9.2 | 7.9 | 3.1 | 9.2 | 10.6 | 6.8 | 6.2 | 892 | 163.7 | 77.1 | ND | ND | ND | ND | ND | ND | ND | ND |
| | Ubc Cre LepR lp/lp | 63.1 | F | 3 | 17.6 | 31.3 | 7.4 | 8.3 | 4.6 | 14.7 | 20.3 | 24.9 | 12.4 | 2203 | 197.1 | 76.8 | ND | ND | ND | ND | ND | ND | ND | ND |
| | Ubc Cre LepR lp/lp | 64.1 | F | 4 | 17.3 | 32.9 | 7.7 | 10.3 | 4.3 | 17.9 | 26.6 | 21.9 | 13.3 | 2283 | 253.1 | 112.3 | ND | ND | ND | ND | ND | ND | ND | ND |
| | Ubc Cre LepR lp/lp | 64.2 | F | 5 | 17.8 | 30.9 | 6.7 | 9.2 | 4.0 | 14.3 | 17.9 | 23.5 | 9.8 | 2000 | 73.9 | 43.2 | ND | ND | ND | ND | ND | ND | ND | ND |
| | Ubc Cre LepR lp/lp | 22.1Bl | M | 6 | 22.4 | 35.0 | 11.3 | 7.3 | 8.6 | 17.0 | 16.2 | 15.8 | 9.9 | 1692 | 353.7 | 615.0 | 11.3 | 12.2 | 12.5 | 7.1 | 6.1 | 8.7 | 9758 | 1878 |
| | Ubc Cre LepR lp/lp | 22.3Bl | M | 7 | 21.5 | 39.0 | 8.2 | 10.3 | 6.2 | 14.1 | 15.3 | 12.5 | 8.4 | 1418 | 477.3 | 490.0 | 11.7 | 14.4 | 13.1 | 7.5 | 7.8 | 9.0 | 10838 | 2090 |
| | Ubc Cre LepR lp/lp | 22.4Bl | M | 8 | 22.5 | 34.9 | 8.9 | 10.2 | 6.7 | 13.9 | 15.1 | 13.3 | 8.8 | 1464 | 1383.6 | 487.9 | 13.6 | 16.0 | 14.8 | 14.4 | 16.4 | 15.4 | 16553 | 1877 |
| | Ubc Cre LepR lp/lp | 22.5Bl | M | 9 | 24.7 | 40.0 | 9.3 | 9.5 | 7.0 | 26.3 | HI | 23.6 | 18.9 | 2702 | 840.2 | 448.0 | 11.3 | 12.7 | 14.1 | 14.7 | 12.8 | 12.5 | 14618 | 2163 |
| | Ubc Cre LepR lp/lp | 24.1Bl | M | 10 | 19.1 | 34.4 | 8.4 | 9.9 | 7.1 | HI | HI | 27.6 | 12.4 | 2710 | 1419.9 | 432.7 | 9.1 | 12.9 | 6.2 | 6.1 | 7.1 | 9.6 | 8273 | 1678 |
| | Ubc Cre LepR lp/lp | 24.2Bl | M | 11 | 18.6 | 27.2 | 9.2 | 8.6 | 6.6 | 11.9 | 9.1 | 8.1 | 6.2 | 979 | 439.7 | 193.9 | 13.3 | 13.5 | 9.7 | 11.3 | 12.2 | 9.2 | 12030 | 1086 |
| | Ubc Cre LepR lp/lp | 24.3Bl | M | 12 | 19.7 | 32.6 | 12.0 | 10.6 | 5.5 | 22.2 | HI | HI | 14.3 | 2683 | 424.2 | 238.1 | 13.1 | 15.9 | 9.6 | 7.1 | 8.8 | 7.7 | 10073 | 1777 |
| | Average | | | | 20.2 | 33.3 | 9.1 | 9.3 | 5.7 | 16.6 | 17.0 | 17.4 | 10.6 | 1887.5 | 526.5 | 275.6 | 11.9 | 13.9 | 11.4 | 9.7 | 10.2 | 10.3 | 11734.7 | 1792.7 |
| | SEM | | | | 0.7 | 1.1 | 0.5 | 0.3 | 0.5 | 1.5 | 1.8 | 2.2 | 1.1 | 186.0 | 130.8 | 59.2 | 0.6 | 0.6 | 1.2 | 1.4 | 1.4 | 1.0 | 1102.7 | 133.9 |
| | p-value | | | | 0.44 | 8.65E-08 | 0.70 | 0.007 | 0.003 | 0.36 | 0.19 | 0.008 | 0.0007 | 0.006 | 0.048 | 0.01 | 0.046 | 0.02 | 0.01 | 0.04 | 0.0098 | 0.001 | 0.005 | 0.00007 |

**Week 5**

| | Group | ID Number | Sex | # | Initial Wt (g) | Final Wt (g) | Init. BG (mmol/l) | Final BG (mmol/l) | Fast BG (mmol/l) | 15 min BG (mmol/l) | 30 min BG (mmol/l) | 60 min BG (mmol/l) | 120 min BG (mmol/l) | AUC (mmol/l x min) | Fed Insulin (pmol/l) | Fasting Insulin (pmol/l) | 4 hr Fast BG (mmol/l) | 0 min BG (mmol/l) | 15 min BG (mmol/l) | 30 min BG (mmol/l) | 45 min BG (mmol/l) | 60 min BG (mmol/l) | AUC (% initial x min) | Gonad Fat (g) |
|---|---|---|---|---|---|---|---|---|---|---|---|---|---|---|---|---|---|---|---|---|---|---|---|---|
| Control | B6.129 | 974.2 | F | 1 | 14.4 | 17.0 | 7.6 | 7.6 | 2.4 | 20.9 | 23.8 | 9.1 | 4.1 | 1397 | 127.9 | 71.8 | ND | ND | ND | ND | ND | ND | ND | ND |
| | B6.129 | 980.1 | F | 2 | 19.7 | 19.4 | 6.4 | 7.7 | 2.4 | 14.8 | 9.8 | 7.2 | 4.5 | 922 | 114.5 | 79.4 | ND | ND | ND | ND | ND | ND | ND | ND |
| | B6.129 | 980.2 | F | 3 | 17.9 | 17.9 | 7.0 | 6.2 | 2.4 | 12.4 | 10.6 | 6.9 | 3.6 | 864 | 204.2 | 54.8 | ND | ND | ND | ND | ND | ND | ND | ND |
| | B6.129 | 980.3 | F | 4 | 18.4 | 17.2 | 7.6 | 6.1 | 2.1 | 21.3 | 18.3 | 10.1 | 4.8 | 1346 | 95.2 | 57.3 | ND | ND | ND | ND | ND | ND | ND | ND |
| | B6.129 | 981.2 | F | 5 | 16.6 | 18.1 | 4.7 | 7.2 | 2.3 | 24.2 | 14.2 | 7.8 | 4.2 | 1370 | 55.3 | 133.6 | ND | ND | ND | ND | ND | ND | ND | ND |
| | B6.129 | 981.4 | F | 6 | 17.1 | 16.6 | 6.0 | 6.8 | 3.1 | 17.8 | 15.3 | 12.9 | 5.2 | 1178 | 71.3 | 364.3 | ND | ND | ND | ND | ND | ND | ND | ND |
| | Average | | | | 17.3 | 17.7 | 6.5 | 6.9 | 2.4 | 18.6 | 15.4 | 9.0 | 4.4 | 1179.5 | 111.4 | 126.9 | | | | | | | | |
| | SEM | | | | 0.7 | 0.4 | 0.5 | 0.3 | 0.1 | 1.8 | 2.1 | 0.9 | 0.2 | 96.0 | 21.5 | 48.9 | | | | | | | | |
| LepR KO | B6.129 | 974.1 | F | 1 | 14.8 | 40.9 | 5.6 | 8.6 | 4.8 | 20.3 | 21.2 | HI | HI | 2901 | 1105.0 | 526.9 | ND | ND | ND | ND | ND | ND | ND | ND |
| | B6.129 | 974.4 | F | 2 | 17.7 | 41.7 | 6.6 | 7.8 | 3.4 | 25.9 | HI | HI | 23.8 | 3003 | 691.3 | 390.5 | ND | ND | ND | ND | ND | ND | ND | ND |
| | B6.129 | 974.5 | F | 3 | 15.4 | 38.0 | 7.6 | 7.4 | 3.8 | 10.2 | 12.8 | 24.7 | HI | 2413 | 979.2 | 420.3 | ND | ND | ND | ND | ND | ND | ND | ND |
| | B6.129 | 978.1 | F | 4 | 17.4 | 40.5 | 5.6 | 6.8 | 4.2 | 25.9 | HI | HI | HI | 3129 | 1623.8 | 405.5 | ND | ND | ND | ND | ND | ND | ND | ND |
| | B6.129 | 978.5 | F | 5 | 16.7 | 45.5 | 5.2 | 6.3 | 4.1 | 14.6 | HI | HI | HI | 2958 | 735.4 | 247.4 | ND | ND | ND | ND | ND | ND | ND | ND |
| | B6.129 | 981.1 | F | 6 | 18.0 | 42.1 | 4.8 | 5.4 | 3.9 | 19.5 | 20.2 | 9.1 | 4.4 | 1320 | 691.5 | 851.9 | ND | ND | ND | ND | ND | ND | ND | ND |
| | B6.129 | 981.3 | F | 7 | 19.4 | 43.5 | 5.4 | 9.1 | 4.2 | 26.2 | HI | HI | 23.3 | 2997 | 800.3 | 575.1 | ND | ND | ND | ND | ND | ND | ND | ND |
| | Average | | | | 17.1 | 41.7 | 5.8 | 7.3 | 4.1 | 20.4 | 18.1 | 16.9 | 17.2 | 2674.4 | 946.6 | 488.2 | | | | | | | | |
| | SEM | | | | 0.6 | 0.9 | 0.4 | 0.5 | 0.2 | 2.4 | 2.6 | 7.8 | 6.4 | 241.7 | 127.3 | 72.4 | | | | | | | | |
| | p-value | | | | 0.76 | 1.16E-10 | 0.24 | 0.49 | 0.00001 | 0.57 | 0.47 | 0.10 | 0.02 | 0.0002 | 0.00009 | 0.002 | | | | | | | | |
