# Supplementary material for: Extreme obesity induces massive beta cell expansion in mice through self-renewal and does not alter the beta cell lineage
Source: Diabetologia. 2016 Mar 22;59:1231–41. doi: 10.1007/s00125-016-3922-7 (PMC4869735; doi:10.1007/s00125-016-3922-7)
Supplement: Supplementary file 5 — (PDF 41 kb) [file 125_2016_3922_MOESM5_ESM.pdf]

ESM Table 3. Breakdown of sexes for experiments performed in Figure 1. Control and LepR KO groups divided by sex with the sample number (n-value) for each experiment listed. For figure I & j, values are listed as week 3, week 5.

	**Control (n-value)**		**LepR KO (n-value)**	
	**Males**	**Females**	**Males**	**Females**
**Figure 1b**	0	7	0	7
**Figure 1c**	0	7	0	7
**Figure 1d**	0	6	0	6
**Figure 1e**	1	4	2	3
**Figure 1f**	0	7	0	7
**Figure 1g**	3	0	7	0
**Figure 1h**	3	0	7	0
**Figure 1i**	3, 0	4, 6	9, 0	3, 7
**Figure 1j**	4, 0	2, 6	9, 0	3, 7
**Figure 1k**	3	0	3	0
**Figure 1l**	3	0	3	0
**Figure 1m**	3	0	3	0
**Figure 1n**	3	0	3	0
**Figure 1o**	3	0	0	3
**Figure 1p**	3	0	0	3
**Figure 1q**	3	0	0	3
