# Supplementary material for: Extreme obesity induces massive beta cell expansion in mice through self-renewal and does not alter the beta cell lineage
Source: Diabetologia. 2016 Mar 22;59:1231–41. doi: 10.1007/s00125-016-3922-7 (PMC4869735; doi:10.1007/s00125-016-3922-7)
Supplement: Supplementary file 6 — (PDF 69 kb) [file 125_2016_3922_MOESM6_ESM.pdf]

ESM Table 4. Individual morphometric data from LepR lp/lp and Ubc Cre LepR lp/lp mice. Pancreas mass (g), total number of slides and tissue sections analyzed, pancreas and insulin area (mm2), beta cell area (% of total pancreas area), and beta cell mass (mg) for the head, tail, and total pancreas. Measurements were made in three different cohorts of LepR lp/lp and Ubc Cre LepR lp/lp mice at 3 and 5 weeks after tamoxifen initiation.

Week 3

					Head Pancreas							Tail Pancreas							Total Pancreas						
	Group	ID Number	Sex	#	Pancreas Mass (mg)	Number of Slides Analyzed	Number of Depths Analyzed	Pancreas Area (mm2)	Insulin Area (mm2)	Beta Cell Area (% Total)	Beta Cell Mass (mg)	Pancreas Mass (mg)	Number of Slides Analyzed	Number of Depths Analyzed	Pancreas Area (mm2)	Insulin Area (mm2)	Beta Cell Area (% Total)	Beta Cell Mass (mg)	Pancreas Mass (mg)	Number of Slides Analyzed	Number of Depths Analyzed	Pancreas Area (um2)	Insulin Area (um2)	Beta Cell Area (% Total)	Beta Cell Mass (mg)
Control	LepR lp/lp	63.2Bl	F	1	88	2	10	145.1	0.78	0.54	0.47	92	2	8	166.5	1.21	0.73	0.67	180	4	18	311.5	1.99	0.64	1.15
	LepR lp/lp	63.3Bl	F	2	121	2	7	150.9	1.06	0.70	0.85	100	2	8	157.8	1.03	0.65	0.65	221	4	15	308.7	2.09	0.68	1.50
	LepR lp/lp	63.4Bl	F	3	65	2	7	105.7	0.48	0.45	0.30	90	2	7	150.4	0.65	0.43	0.39	155	4	14	256.1	1.13	0.44	0.68
	LepR lp/lp	64.3Bl	F	4	58	2	7	88.7	0.54	0.61	0.35	69	2	8	120.0	0.80	0.67	0.46	127	4	15	208.7	1.34	0.64	0.81
	LepR lp/lp	62.3Bl	M	5	111	2	7	182.3	0.49	0.27	0.30	117	2	8	188.7	1.27	0.67	0.79	228	4	15	371.0	1.76	0.47	1.08
	LepR lp/lp	22.2Bl	M	6	85	2	7	177.9	1.09	0.61	0.52	130	2	8	314.2	1.55	0.49	0.64	215	4	15	492.1	2.64	0.54	1.15
	LepR lp/lp	24.4Bl	M	7	93	2	8	175.6	0.53	0.30	0.28	143	2	8	293.5	1.35	0.46	0.66	236	4	16	469.1	1.88	0.40	0.95
	LepR lp/lp	25.1Bl	M	8	74	2	8	166.4	0.59	0.36	0.26	117	2	8	317.8	1.81	0.57	0.66	191	4	16	484.2	2.40	0.50	0.95
	Average				86.9	2.0	7.6	149.1	0.70	0.48	0.42	107.3	2.0	7.9	213.6	1.21	0.58	0.61	194.1	4.0	15.5	362.7	1.90	0.54	1.05
	SEM				7.6			12.3	0.09	0.06	0.07	8.5			28.7	0.13	0.04	0.05	13.6			38.6	0.18	0.04	0.09
LepR KO	Ubc Cre LepR lp/lp	63.1Bl	F	1	72	2	9	145.4	1.79	1.23	0.89	95	2	7	138.7	1.57	1.13	1.08	167	4	16	284.0	3.36	1.18	1.98
	Ubc Cre LepR lp/lp	64.1Bl	F	2	80	2	7	171.0	1.49	0.87	0.70	111	2	10	201.7	3.52	1.75	1.94	191	4	17	372.8	5.01	1.34	2.57
	Ubc Cre LepR lp/lp	64.2Bl	F	3	101	2	7	194.4	1.39	0.71	0.72	115	2	10	184.7	2.01	1.09	1.25	216	4	17	379.2	3.40	0.90	1.94
	Ubc Cre LepR lp/lp	62.1Bl	M	4	110	2	7	127.6	1.07	0.84	0.92	131	2	7	185.6	1.83	0.99	1.29	241	4	14	313.2	2.90	0.93	2.23
	Ubc Cre LepR lp/lp	62.2Bl	M	5	94	2	9	193.5	1.58	0.82	0.77	119	2	8	181.7	1.73	0.95	1.13	213	4	17	375.2	3.31	0.88	1.88
	Ubc Cre LepR lp/lp	22.1Bl	M	6	133	2	8	259.6	1.89	0.73	0.97	165	2	8	356.7	3.24	0.91	1.50	298	4	16	616.3	5.13	0.83	2.48
	Ubc Cre LepR lp/lp	22.3Bl	M	7	116	2	8	227.9	1.42	0.62	0.72	138	2	8	350.7	4.29	1.22	1.69	254	4	16	578.6	5.71	0.99	2.51
	Ubc Cre LepR lp/lp	22.4Bl	M	8	114	2	8	255.8	1.55	0.61	0.69	137	2	8	298.9	3.26	1.09	1.49	251	4	16	554.6	4.81	0.87	2.18
	Ubc Cre LepR lp/lp	22.5Bl	M	9	140	2	8	307.8	2.92	0.95	1.33	149	2	8	369.1	3.97	1.08	1.60	289	4	16	676.9	6.89	1.02	2.94
	Ubc Cre LepR lp/lp	24.1Bl	M	10	ND	ND	ND	ND	ND	ND	ND	ND	ND	ND	ND	ND	ND	ND	ND	ND	ND	ND	ND	ND	ND
	Ubc Cre LepR lp/lp	24.2Bl	M	11	ND	ND	ND	ND	ND	ND	ND	ND	ND	ND	ND	ND	ND	ND	ND	ND	ND	ND	ND	ND	ND
	Ubc Cre LepR lp/lp	24.3Bl	M	12	ND	ND	ND	ND	ND	ND	ND	ND	ND	ND	ND	ND	ND	ND	ND	ND	ND	ND	ND	ND	ND
	Average				106.7	2.0	7.9	209.2	1.68	0.82	0.86	128.9	2.0	8.2	252.0	2.82	1.13	1.44	235.6	4.0	16.1	461.2	4.50	0.99	2.30
	SEM				7.5			19.5	0.17	0.06	0.07	7.1			30.2	0.35	0.08	0.09	14.4			48.4	0.45	0.06	0.12
	p-value				0.09			0.02	0.0002	0.001	0.0005	0.07			0.38	0.001	3.88E-05	1.48E-06	0.055			0.14	0.0001	8.49E-06	4.34E-07

Week 5

					Head Pancreas							Tail Pancreas							Total Pancreas						
	Group	ID Number	Sex	#	Pancreas Mass (mg)	Number of Slides Analyzed	Number of Depths Analyzed	Pancreas Area (mm2)	Insulin Area (mm2)	Beta Cell Area (% Total)	Beta Cell Mass (mg)	Pancreas Mass (mg)	Number of Slides Analyzed	Number of Depths Analyzed	Pancreas Area (mm2)	Insulin Area (mm2)	Beta Cell Area (% Total)	Beta Cell Mass (mg)	Pancreas Mass (mg)	Number of Slides Analyzed	Number of Depths Analyzed	Pancreas Area (mm2)	Insulin Area (mm2)	Beta Cell Area (% Total)	Beta Cell Mass (mg)
Control	LepR lp/lp	974.2	F	1	84	2	6	71.9	0.40	0.56	0.47	80	2	6	103.9	1.11	1.07	0.85	164	4	12	175.8	1.51	0.86	1.41
	LepR lp/lp	980.1	F	3	68	2	10	149.8	0.54	0.36	0.25	68	2	7	138.2	0.81	0.59	0.40	136	4	17	288.0	1.35	0.47	0.64
	LepR lp/lp	980.2	F	4	54	2	10	134.4	0.42	0.31	0.17	75	2	10	186.7	1.12	0.60	0.45	129	4	20	321.1	1.54	0.48	0.62
	LepR lp/lp	980.3	F	5	53	2	6	115.1	0.51	0.44	0.23	70	2	8	144.6	0.93	0.64	0.45	123	4	14	259.7	1.44	0.56	0.68
	LepR lp/lp	981.2	F	6	70	2	8	152.5	0.49	0.32	0.22	79	2	10	174.7	1.13	0.65	0.51	149	4	18	327.3	1.62	0.50	0.74
	LepR lp/lp	981.4	F	7	66	2	7	141.2	0.63	0.45	0.29	81	2	10	217.0	1.47	0.68	0.55	147	4	17	358.2	2.10	0.59	0.86
	Average				65.8	2.0	7.8	127.5	0.50	0.41	0.27	75.5	2.0	8.5	160.9	1.10	0.70	0.54	141.3	4.0	16.3	288.3	1.60	0.57	0.83
	SEM				4.7			12.4	0.03	0.04	0.04	2.2			16.4	0.09	0.07	0.07	6.1			26.4	0.11	0.06	0.12
LepR KO	Ubc Cre LepR lp/lp	974.1	F	1	52	2	4	77.0	1.16	1.51	0.78	81	2	6	127.6	3.30	2.59	2.10	133	4	10	204.5	4.46	2.18	2.90
	Ubc Cre LepR lp/lp	974.4	F	2	86	2	6	128.5	1.95	1.52	1.31	99	2	6	116.1	3.04	2.62	2.59	185	4	12	244.6	4.99	2.04	3.77
	Ubc Cre LepR lp/lp	974.5	F	3	79	2	6	103.7	1.30	1.25	0.99	85	2	6	72.9	1.13	1.55	1.32	164	4	12	176.6	2.43	1.38	2.26
	Ubc Cre LepR lp/lp	978.1	F	4	86	2	5	105.2	1.35	1.28	1.10	117	2	6	109.6	2.68	2.45	2.87	203	4	11	214.8	4.03	1.88	3.81
	Ubc Cre LepR lp/lp	978.5	F	5	75	2	6	135.3	2.23	1.65	1.24	112	2	6	152.0	2.43	1.60	1.79	187	4	12	287.3	4.66	1.62	3.03
	Ubc Cre LepR lp/lp	981.1	F	6	94	2	8	178.2	1.99	1.12	1.05	112	2	8	247.6	4.41	1.78	1.99	206	4	16	425.8	6.40	1.50	3.10
	Ubc Cre LepR lp/lp	981.3	F	7	79	2	8	139.6	1.64	1.17	0.93	113	2	6	162.3	2.70	1.66	1.88	192	4	14	301.9	4.34	1.44	2.76
	Average				78.7	2.0	6.1	123.9	1.66	1.36	1.06	102.7	2.0	6.3	141.1	2.81	2.04	2.08	181.4	4.0	12.4	265.1	4.47	1.72	3.09
	SEM				5.0			12.3	0.15	0.08	0.07	5.5			20.9	0.37	0.19	0.19	9.6			31.7	0.45	0.12	0.21
	p-value				0.09			0.84	2.85E-05	3.98E-07	1.31E-06	0.001			0.48	0.0016	6.31E-05	2.34E-05	0.006			0.59	0.0001	5.57E-06	2.22E-06
