# Supplementary material for: Extreme obesity induces massive beta cell expansion in mice through self-renewal and does not alter the beta cell lineage
Source: Diabetologia. 2016 Mar 22;59:1231–41. doi: 10.1007/s00125-016-3922-7 (PMC4869735; doi:10.1007/s00125-016-3922-7)
Supplement: Supplementary file 7 — (PDF 64 kb) [file 125_2016_3922_MOESM7_ESM.pdf]

ESM Table 5. Individual morphometric data from LepR lp/lp and Ubc Cre LepR lp/lp mice. Beta cell size (μm2), cross-sectional beta cell area per islet (μm2), total islet number, and the number of islets within each size category. Measurements were made in three different cohorts of LepR lp/lp and Ubc Cre LepR lp/lp mice at 3 and 5 weeks after tamoxifen initiation.

Week 3

					Head Pancreas							Tail Pancreas							Total Pancreas						
					Beta Cell Size (μm2)	Cross-sectional Beta Cell Area per Islet (μm2)	Total Islet Number	Number of Islet per Islet Size (um2)				Beta Cell Size (μm2)	Cross-sectional Beta Cell Area per Islet (μm2)	Total Islet Number	Number of Islet per Islet Size (um2)				Beta Cell Size (μm2)	Cross-sectional Beta Cell Area per Islet (μm2)	Total Islet Number	Number of Islet per Islet Size (um2)			
	Group	ID Number	Sex	#				<1000 μm2	1001 - 5000 μm2	5001 - 10000 μm2	>10000 μm2				<1000 μm2	1001 - 5000 μm2	5001 - 10000 μm2	>10000 μm2				<1000 μm2	1001 - 5000 μm2	5001 - 10000 μm2	>10000 μm2
Control	LepR lp/lp	63.2Bl	F	1	146.1	1511.1	325	234	70	13	8	133.8	2983.5	424	276	84	26	38	138.5	2344.6	749	510.0	154.00	39	46
	LepR lp/lp	63.3Bl	F	2	152.2	1571.0	499	384	74	18	23	166.0	2048.6	591	428	109	24	30	159.8	1830.0	1090	812	183	42	53
	LepR lp/lp	63.4Bl	F	3	117.1	1611.0	655	513	101	16	25	142.7	1626.7	724	550	114	32	28	130.2	1619.3	1379	1063	215	48	53
	LepR lp/lp	64.3Bl	F	4	116.4	1971.5	243	170	47	13	13	147.8	2084.4	310	200	71	24	15	133.3	2034.8	553	370	118	37	28
	LepR lp/lp	62.3Bl	M	5	122.1	1915.8	282	199	62	5	16	170.0	2103.8	380	274	65	24	17	147.0	2023.7	662	473	127	29	33
	LepR lp/lp	22.2Bl	M	6	122.1	3421.0	319	196	67	23	33	125.1	2656.5	582	409	108	22	43	124.0	2927.2	901	605	175	45	76
	LepR lp/lp	24.4Bl	M	7	126.1	2438.4	219	140	49	18	12	120.7	3008.2	450	293	93	32	32	122.3	2821.7	669	433	142	50	44
	LepR lp/lp	25.1Bl	M	8	117.7	2018.8	294	211	54	16	13	130.1	2533.3	713	505	126	34	48	123.0	2383.1	1007	716	180	50	61
	Average				127.5	2057.3	354.5	255.9	65.5	15.3	17.9	142.0	2380.6	521.8	366.9	96.3	27.3	31.4	134.8	2248.0	876.3	622.8	161.8	42.5	47.6
	SEM				4.9	222.3	52.3	44.8	6.1	1.8	3.0	6.5	174.2	54.4	44.0	7.6	1.6	4.1	4.7	162.8	96.9	81.7	11.5	2.6	5.4
LepR KO	Ubc Cre LepR lp/lp	63.1Bl	F	1	177.0	2992.3	359	235	72	21	31	199.9	3823.6	479	305	97	27	50	188.2	3467.4	838	540	169	48	81
	Ubc Cre LepR lp/lp	64.1Bl	F	2	123.6	2294.8	689	491	128	31	39	174.7	3586.9	481	307	91	32	51	145.8	2826.0	1170	798	219	63	90
	Ubc Cre LepR lp/lp	64.2Bl	F	3	130.7	2598.3	635	453	128	16	38	149.1	2933.8	534	370	103	26	35	139.7	2744.9	1169	823	231	42	73
	Ubc Cre LepR lp/lp	62.1Bl	M	4	219.7	2824.1	528	320	142	32	34	171.6	3844.2	916	597	189	46	84	194.6	3471.2	1444	917	331	78	118
	Ubc Cre LepR lp/lp	62.2Bl	M	5	205.1	3141.7	353	242	70	13	28	172.8	3864.9	520	326	117	19	58	186.8	3572.5	873	568	187	32	86
	Ubc Cre LepR lp/lp	22.1Bl	M	6	157.8	4269.5	442	297	76	23	46	151.0	4639.3	699	423	141	42	93	154.8	4496.0	1141	720	217	65	139
	Ubc Cre LepR lp/lp	22.3Bl	M	7	157.0	3380.6	420	249	112	22	37	147.2	7087.7	605	346	111	41	107	150.1	5568.7	1025	595	223	63	144
	Ubc Cre LepR lp/lp	22.4Bl	M	8	139.5	2646.7	587	394	123	32	38	128.2	4382.4	744	472	151	33	88	131.8	3616.9	1331	866	274	65	126
	Ubc Cre LepR lp/lp	22.5Bl	M	9	149.0	4110.1	711	441	160	41	69	156.4	5198.8	764	438	168	53	105	151.8	4674.0	1475	879	328	94	174
	Ubc Cre LepR lp/lp	24.1Bl	M	10	ND	ND	ND	ND	ND	ND	ND	ND	ND	ND	ND	ND	ND	ND	ND	ND	ND	ND	ND	ND	ND
	Ubc Cre LepR lp/lp	24.2Bl	M	11	ND	ND	ND	ND	ND	ND	ND	ND	ND	ND	ND	ND	ND	ND	ND	ND	ND	ND	ND	ND	ND
	Ubc Cre LepR lp/lp	24.3Bl	M	12	ND	ND	ND	ND	ND	ND	ND	ND	ND	ND	ND	ND	ND	ND	ND	ND	ND	ND	ND	ND	ND
	Average				162.2	3139.8	524.9	346.9	112.3	25.7	40.0	161.2	4373.5	638.0	398.2	129.8	35.4	74.6	160.4	3826.4	1162.9	745.1	242.1	61.1	114.6
	SEM				10.9	225.1	46.0	33.2	10.9	3.0	4.0	6.9	402.3	50.5	31.8	11.4	3.6	8.8	7.7	305.9	75.8	48.2	19.1	6.2	11.4
	p-value				0.01	0.004	0.03	0.12	0.002	0.01	0.0006	0.06	0.0006	0.14	0.57	0.03	0.07	0.0007	0.015	0.0005	0.03	0.20	0.003	0.02	0.0002

Week 5

					Head Pancreas							Tail Pancreas							Total Pancreas						
					Beta Cell Size (μm2)	Cross-sectional Beta Cell Area per Islet (μm2)	Total Islet Number	Number of Islet per Islet Size (um2)				Beta Cell Size (μm2)	Cross-sectional Beta Cell Area per Islet (μm2)	Total Islet Number	Number of Islet per Islet Size (um2)				Beta Cell Size (μm2)	Cross-sectional Beta Cell Area per Islet (μm2)	Total Islet Number	Number of Islet per Islet Size (um2)			
	Group	ID Number	Sex	#				<1000 μm2	1001 - 5000 μm2	5001 - 10000 μm2	>10000 μm2				<1000 μm2	1001 - 5000 μm2	5001 - 10000 μm2	>10000 μm2				<1000 μm2	1001 - 5000 μm2	5001 - 10000 μm2	>10000 μm2
Control	LepR lp/lp	974.2	F	1	101.8	1911.7	201.17	142	48	12	9	127.2	3458.3	321	201	68	23	29	118.4	2844.9	532	343	116	35	38
	LepR lp/lp	980.1	F	3	113.1	1598.4	340	243	66	20	11	103.7	3061.8	267	167	59	17	24	107.1	2242.1	607	410	125	37	35
	LepR lp/lp	980.2	F	4	88.0	1581.8	264	185	59	9	11	111.9	2772.9	218	125	47	20	26	103.3	2302.2	1012	504	248	91	169
	LepR lp/lp	980.3	F	5	97.8	1591.4	322	227	70	14	11	107.4	2511.1	371	223	92	33	23	102.2	2083.8	693	450	162	47	34
	LepR lp/lp	981.2	F	6	ND	1973.9	246	179	39	16	12	ND	2853.9	397	246	98	19	34	ND	2517.2	643	425	137	35	46
	LepR lp/lp	981.4	F	7	ND	1759.0	358	252	77	12	17	ND	3040.8	485	294	114	33	44	ND	2496.5	843	546	191	45	61
	Average				100.2	1736.0	288.5	204.7	59.8	13.8	11.8	112.5	2949.8	343.2	209.3	79.7	24.2	30.0	107.8	2414.4	721.7	446.3	163.17	48.33	63.83
	SEM				5.2	71.1	24.9	17.5	5.8	1.6	1.1	5.2	130.7	39.1	24.3	10.5	2.9	3.2	3.7	108.7	71.9	29.3	20.28	8.79	21.43
LepR KO	Ubc Cre LepR lp/lp	974.1	F	1	119.9	4455.9	260	138	69	25	28	119.8	6898.8	479	256	116	36	71	119.8	6039.3	739	394	185	61	99
	Ubc Cre LepR lp/lp	974.4	F	2	136.6	3752.7	519	288	140	39	52	124.4	5910.1	514	268	126	51	69	130.3	4826.2	1033	556	266	90	121
	Ubc Cre LepR lp/lp	974.5	F	3	103.0	2984.0	436	266	104	35	31	126.9	4155.0	272	170	66	14	22	116.4	3433.9	708	436	170	49	53
	Ubc Cre LepR lp/lp	978.1	F	4	112.5	4197.1	322	192	83	16	31	106.3	7102.4	378	199	89	27	63	110.1	5766.0	700	391	172	43	94
	Ubc Cre LepR lp/lp	978.5	F	5	98.6	3669.2	608	338	164	48	58	115.7	5332.3	455	223	135	28	69	105.1	4381.1	1063	561	299	76	127
	Ubc Cre LepR lp/lp	981.1	F	6	152.7	3490.9	570	327	148	44	51	145.0	5050.8	873	528	194	41	110	147.9	4434.6	1443	855	342	85	161
	Ubc Cre LepR lp/lp	981.3	F	7	ND	3828.4	1115	247	114	28	41	ND	5956.7	453	232	117	35	69	ND	4920.2	1568	479	231	63	110
	Average				120.5	3768.3	547.1	256.6	117.4	33.6	41.7	123.0	5772.3	489.1	268.0	120.4	33.1	67.6	121.6	4828.7	1036.3	524.6	237.86	66.71	109.29
	SEM				8.5	180.1	106.1	27.2	13.2	4.3	4.6	5.3	391.0	70.7	45.1	15.1	4.4	9.7	6.3	332.9	134.4	61.0	25.43	6.70	12.57
	p-value				0.11	8.6E-07	0.05	0.15	0.003	0.002	0.0001	0.22	5.1E-05	0.11	0.30	0.06	0.13	0.005	0.14	4.9E-05	0.08	0.30	0.047	0.12	0.08
