# Supplementary material for: Extreme obesity induces massive beta cell expansion in mice through self-renewal and does not alter the beta cell lineage
Source: Diabetologia. 2016 Mar 22;59:1231–41. doi: 10.1007/s00125-016-3922-7 (PMC4869735; doi:10.1007/s00125-016-3922-7)
Supplement: Supplementary file 8 — (PDF 81 kb) [file 125_2016_3922_MOESM8_ESM.pdf]

ESM Table 6. Individual β-cell proliferation data from LepR lp/lp and Ubc Cre LepR lp/lp mice. Total β-cells counted, BrdU+ β-cells counted (number) and the percentage of total β-cells, EdU+ β-cells counted (number) and the percentage of total β-cells, actual BrdU+ EdU+ β-cells counted (number) and the percentage of total β-cells,the predicted number of BrdU+ EdU+ β-cells (percent of total β-cells), and the actual BrdU+ EdU+ β-cells as a percentage of the predicted BrdU+ EdU+ β-cells for the head, tail, and total pancreas. Measurements were made in three different cohorts of LepR lp/lp and Ubc Cre LepR lp/lp mice at 3 and 5 weeks after tamoxifen initiation.

Week 3

					Head Pancreas									Tail Pancreas									Total Pancreas								
	Group	ID Number	Sex	#	β-Cells	BrdU+ β-Cells	BrdU+ β-Cells (%)	EdU+ β-Cells	EdU+ β-Cells (%)	Actual BrdU+ EdU+ β-Cells	Actual BrdU+ EdU+ β-Cells (%)	Predicted BrdU+ EdU+ β-Cells (%)	Actual BrdU+ EdU+ β-Cells (% Predicted)	β-Cells	BrdU+ β-Cells	BrdU+ β-Cells (%)	EdU+ β-Cells	EdU+ β-Cells (%)	Actual BrdU+ EdU+ β-Cells	Actual BrdU+ EdU+ β-Cells (%)	Predicted BrdU+ EdU+ β-Cells (%)	Actual BrdU+ EdU+ β-Cells (% Predicted)	β-Cells	BrdU+ β-Cells	BrdU+ β-Cells (%)	EdU+ β-Cells	EdU+ β-Cells (%)	Actual BrdU+ EdU+ β-Cells	Actual BrdU+ EdU+ β-Cells (%)	Predicted BrdU+ EdU+ β-Cells (%)	Actual BrdU+ EdU+ β-Cells (% Predicted)
Control	LepR lp/lp	63.2Bl	F	1	2178	306	14.05	109	5.00	1	0.05	0.70	6.53	2353	276	11.73	136	5.78	1	0.04	0.68	6.27	4531	582	12.84	245	5.41	2	0.04	0.69	6.36
	LepR lp/lp	63.3Bl	F	2	3825	226	5.91	44	1.15	2	0.05	0.07	76.93	6221	408	6.56	134	2.15	2	0.03	0.14	22.76	10046	634	6.31	178	1.77	4	0.04	0.11	35.61
	LepR lp/lp	63.4Bl	F	3	3505	304	8.67	116	3.31	2	0.06	0.29	19.88	4275	466	10.90	163	3.81	3	0.07	0.42	16.88	7780	770	9.90	279	3.59	5	0.06	0.35	18.11
	LepR lp/lp	64.3Bl	F	4	2350	110	4.68	112	4.77	1	0.04	0.22	19.07	2475	142	5.74	111	4.48	3	0.12	0.26	47.11	4825	252	5.22	223	4.62	4	0.08	0.24	34.34
	LepR lp/lp	62.3Bl	M	5	2845	121	4.25	9	0.32	0	0.00	0.01	0.00	3327	206	6.19	42	1.26	2	0.06	0.08	76.91	6172	327	5.30	51	0.83	2	0.03	0.04	74.02
	LepR lp/lp	22.2Bl	M	6	3287	273	8.31	103	3.13	0	0.00	0.26	0.00	5743	333	5.80	148	2.58	1	0.02	0.15	11.65	9030	606	6.71	251	2.78	1	0.01	0.19	5.94
	LepR lp/lp	24.4Bl	M	7	1527	151	9.89	48	3.14	0	0.00	0.31	0.00	3427	259	7.56	116	3.38	1	0.03	0.26	11.41	4954	410	8.28	164	3.31	1	0.02	0.27	7.37
	LepR lp/lp	25.1Bl	M	8	2463	187	7.59	164	6.66	0	0.00	0.51	0.00	1797	154	8.57	126	7.01	0	0.00	0.60	0.00	4260	341	8.00	290	6.81	0	0.00	0.54	0.00
	Average				2747.5	209.8	7.92	88.1	3.44	0.8	0.02	0.30	15.30	3702.3	280.5	7.88	122.0	3.81	1.6	0.05	0.32	24.12	6449.8	490.3	7.82	210.1	3.64	2.4	0.04	0.31	22.72
	SEM				270.2	28.1	1.12	17.7	0.73	0.3	0.01	0.08	9.30	567.1	41.1	0.82	12.9	0.68	0.4	0.01	0.08	9.05	788.2	64.5	0.91	27.6	0.69	0.6	0.01	0.08	8.72
LepR KO	Ubc Cre LepR lp/lp	63.1Bl	F	1	2057	1039	50.51	904	43.95	100	4.86	22.20	21.90	2231	957	42.90	378	16.94	71	3.18	7.27	43.79	4288	1996	46.55	1282	29.90	171	3.99	13.92	28.66
	Ubc Cre LepR lp/lp	64.1Bl	F	2	2130	896	42.07	281	13.19	26	1.22	5.55	22.00	2777	1027	36.98	379	13.65	31	1.12	5.05	22.12	4907	1923	39.19	660	13.45	57	1.16	5.27	22.04
	Ubc Cre LepR lp/lp	64.2Bl	F	3	2058	1172	56.95	489	23.76	59	2.87	13.53	21.19	1974	908	46.00	499	25.28	67	3.39	11.63	29.19	4032	2080	51.59	988	24.50	126	3.13	12.64	24.72
	Ubc Cre LepR lp/lp	62.1Bl	M	4	2414	1222	50.62	815	33.76	85	3.52	17.09	20.60	1912	838	43.83	650	34.00	91	4.76	14.90	31.94	4326	2060	47.62	1465	33.87	176	4.07	16.13	25.23
	Ubc Cre LepR lp/lp	62.2Bl	M	5	2165	1482	68.45	543	25.08	44	2.03	17.17	11.84	2093	1056	50.45	597	28.52	51	2.44	14.39	16.93	4258	2538	59.61	1140	26.77	95	2.23	15.96	13.98
	Ubc Cre LepR lp/lp	22.1Bl	M	6	3945	2023	51.28	662	16.78	73	1.85	8.61	21.50	3154	1607	50.95	540	17.12	54	1.71	8.72	19.63	7099	3630	51.13	1202	16.93	127	1.79	8.66	20.66
	Ubc Cre LepR lp/lp	22.3Bl	M	7	2995	1277	42.64	883	29.48	71	2.37	12.57	18.86	7139	3210	44.96	1697	23.77	169	2.37	10.69	22.15	10134	4487	44.28	2580	25.46	240	2.37	11.27	21.01
	Ubc Cre LepR lp/lp	22.4Bl	M	8	2326	1260	54.17	381	16.38	35	1.50	8.87	16.96	4928	2059	41.78	710	14.41	39	0.79	6.02	13.15	7254	3319	45.75	1091	15.04	74	1.02	6.88	14.82
	Ubc Cre LepR lp/lp	22.5Bl	M	9	4014	2197	54.73	720	17.94	70	1.74	9.82	17.76	2488	1347	54.14	465	18.69	70	2.81	10.12	27.81	6502	3544	54.51	1185	18.23	140	2.15	9.93	21.68
	Ubc Cre LepR lp/lp	24.1Bl	M	10	ND	ND	ND	ND	ND	ND	ND	ND	ND	ND	ND	ND	ND	ND	ND	ND	ND	ND	ND	ND	ND	ND	ND	ND	ND	ND	ND
	Ubc Cre LepR lp/lp	24.2Bl	M	11	ND	ND	ND	ND	ND	ND	ND	ND	ND	ND	ND	ND	ND	ND	ND	ND	ND	ND	ND	ND	ND	ND	ND	ND	ND	ND	ND
	Ubc Cre LepR lp/lp	24.3Bl	M	12	ND	ND	ND	ND	ND	ND	ND	ND	ND	ND	ND	ND	ND	ND	ND	ND	ND	ND	ND	ND	ND	ND	ND	ND	ND	ND	ND
	Average				2678.2	1396.4	52.38	630.9	24.48	62.6	2.44	12.82	19.18	3188.4	1445.4	45.78	657.2	21.38	71.4	2.51	9.86	25.19	5866.7	2841.9	48.91	1288.1	22.68	134.00	2.43	11.18	21.42
	SEM				264.0	145.9	2.63	73.9	3.30	8.0	0.38	1.75	1.10	583.5	256.8	1.77	135.3	2.31	13.6	0.41	1.15	3.07	685.1	309.4	2.01	177.3	2.36	18.84	0.37	1.28	1.57
	p-value				0.86	1.79E-06	2.18E-10	6.57E-06	3.07E-05	2.77E-06	2.81E-05	6.95E-06	0.67	0.54	0.0007	8.77E-12	0.002	4.94E-06	0.0002	4.41E-05	1.25E-06	0.91	0.58	4.13E-06	1.65E-11	4.55E-05	2.45E-06	9.07E-06	1.94E-05	9.13E-07	0.88

Week 5

					Head Pancreas									Tail Pancreas									Total Pancreas								
	Group	ID Number	Sex	#	β-Cells	BrdU+ β-Cells	BrdU+ β-Cells (%)	EdU+ β-Cells	EdU+ β-Cells (%)	Actual BrdU+ EdU+ β-Cells	Actual BrdU+ EdU+ β-Cells (%)	Predicted BrdU+ EdU+ β-Cells (%)	Actual BrdU+ EdU+ β-Cells (% Predicted)	β-Cells	BrdU+ β-Cells	BrdU+ β-Cells (%)	EdU+ β-Cells	EdU+ β-Cells (%)	Actual BrdU+ EdU+ β-Cells	Actual BrdU+ EdU+ β-Cells (%)	Predicted BrdU+ EdU+ β-Cells (%)	Actual BrdU+ EdU+ β-Cells (% Predicted)	β-Cells	BrdU+ β-Cells	BrdU+ β-Cells (%)	EdU+ β-Cells	EdU+ β-Cells (%)	Actual BrdU+ EdU+ β-Cells	Actual BrdU+ EdU+ β-Cells (%)	Predicted BrdU+ EdU+ β-Cells (%)	Actual BrdU+ EdU+ β-Cells (% Predicted)
Control	LepR lp/lp	974.2	F	1	2420	90	3.72	67	2.77	0	0.00	0.10	0.00	8813	397	4.50	274	3.11	2	0.02	0.14	16.20	11233	487	4.34	341	3.04	2	0.02	0.13	13.53
	LepR lp/lp	980.1	F	3	2098	39	1.86	70	3.34	0	0.00	0.06	0.00	2101	57	2.71	77	3.66	0	0.00	0.10	0.00	4199	96	2.29	147	3.50	0.00	0.00	0.08	0.00
	LepR lp/lp	980.2	F	4	1770	19	1.07	31	1.75	0	0.00	0.02	0.00	2145	36	1.68	39	1.82	0	0.00	0.03	0.00	3915	55	1.40	70	1.79	0.00	0.00	0.03	0.00
	LepR lp/lp	980.3	F	5	1437	12	0.84	8	0.56	0	0.00	0.005	0.00	1988	19	0.96	41	2.06	0	0.00	0.02	0.00	3425	31	0.91	49	1.43	0.00	0.00	0.01	0.00
	LepR lp/lp	981.2	F	6	2254	24	1.06	9	0.40	0	0.00	0.004	0.00	2404	73	3.04	61	2.54	0	0.00	0.08	0.00	4658	97	2.08	70	1.50	0.00	0.00	0.03	0.00
	LepR lp/lp	981.4	F	7	1418	15	1.06	14	0.99	0	0.00	0.01	0.00	2081	52	2.50	58	2.79	0	0.00	0.07	0.00	3499	67	1.91	72	2.06	0.00	0.00	0.04	0.00
	Average				1899.5	33.2	1.60	33.2	1.63	0.0	0.00	0.03	0.00	3255.3	105.7	2.56	91.7	2.66	0.3	0.00	0.07	2.70	5154.8	138.8	2.15	124.8	2.22	0.33	0.00	0.05	2.25
	SEM				173.1	12.0	0.45	11.7	0.49	0.0	0.00	0.02	0.00	1113.0	58.8	0.50	36.9	0.28	0.3	0.00	0.02	2.70	1229.8	70.4	0.48	45.4	0.35	0.33	0.00	0.02	2.25
LepR KO	Ubc Cre LepR lp/lp	974.1	F	1	2400	746	31.08	860	35.83	24	1.00	11.14	8.98	2535	567	22.37	785	30.97	22	0.87	6.93	12.53	4935	1313	26.61	1645	33.33	46.00	0.93	8.87	10.51
	Ubc Cre LepR lp/lp	974.4	F	2	2477	692	27.94	512	20.67	7	0.28	5.77	4.89	2508	495	19.74	454	18.10	8	0.32	3.57	8.93	4985	1187	23.81	966	19.38	15.00	0.30	4.61	6.52
	Ubc Cre LepR lp/lp	974.5	F	3	1772	193	10.89	85	4.80	1	0.06	0.52	10.80	2285	304	13.30	207	9.06	5	0.22	1.21	18.16	4057	497	12.25	292	7.20	6.00	0.15	0.88	16.77
	Ubc Cre LepR lp/lp	978.1	F	4	2412	179	7.42	116	4.81	0	0.00	0.36	0.00	2327	132	5.67	139	5.97	1	0.04	0.34	12.68	4739	311	6.56	255	5.38	1.00	0.02	0.35	5.98
	Ubc Cre LepR lp/lp	978.5	F	5	2010	345	17.16	457	22.74	13	0.65	3.90	16.57	2366	421	17.79	798	33.73	18	0.76	6.00	12.68	4376	766	17.50	1255	28.68	31.00	0.71	5.02	14.11
	Ubc Cre LepR lp/lp	981.1	F	6	2064	280	13.57	60	2.91	2	0.10	0.39	24.57	2005	374	18.65	85	4.24	2	0.10	0.79	12.61	4069	654	16.07	145	3.56	4.00	0.10	0.57	17.16
	Ubc Cre LepR lp/lp	981.3	F	7	2097	463	22.08	286	13.64	16	0.76	3.01	25.34	2014	487	24.18	253	12.56	13	0.65	3.04	21.25	4111	950	23.11	539	13.11	29.00	0.71	3.03	23.28
	Average				2176.0	414.0	18.59	339.4	15.06	9.0	0.41	3.59	13.02	2291.4	397.1	17.39	388.7	16.38	9.9	0.42	3.12	14.12	4467.4	811.1	17.99	728.1	15.81	18.86	0.42	3.33	13.48
	SEM				98.3	86.8	3.33	110.2	4.58	3.4	0.15	1.48	3.63	80.5	54.9	2.35	112.8	4.47	3.1	0.13	0.97	1.57	156.1	137.0	2.68	216.3	4.43	6.37	0.14	1.17	2.36
	p-value				0.18	0.002	0.0007	0.03	0.02	0.03	0.03	0.05	0.007	0.37	0.004	0.0001	0.04	0.02	0.02	0.01	0.01	0.003	0.56	0.002	0.0002	0.03	0.02	0.02	0.02	0.03	0.006
