# Supplementary material for: Extreme obesity induces massive beta cell expansion in mice through self-renewal and does not alter the beta cell lineage
Source: Diabetologia. 2016 Mar 22;59:1231–41. doi: 10.1007/s00125-016-3922-7 (PMC4869735; doi:10.1007/s00125-016-3922-7)
Supplement: Supplementary file 9 — (PDF 55 kb) [file 125_2016_3922_MOESM9_ESM.pdf]

ESM Table 7. Individual physiologic data from Rosa Confetti LepR lp/lp and Ubc Cre Rosa Confetti LepR lp/lp mice. Initial and final body weight (g), gonadal fat weight (g), initial and final blood glucose (mmol/l), glucose tolerance test blood glucose measurements (mmol/l) after overnight fast (mmol/l) and at time 15, 30, 60, and 120 min followed by the area under the curve (AUC; mmol/l x min). Measurements were made in three different cohorts of Rosa Confetti LepR lp/lp and Ubc Cre Rosa Confetti LepR lp/lp mice at 1, 3, and 5 weeks after tamoxifen initiation.

					1 month washout										
	Group	ID Number	Sex	#	Initial Wt (g)	Final Wt (g)	Gonad Fat (g)	Init. BG (mmol/l)	Final BG (mmol/l)	Fast BG (mmol/l)	15 min BG (mmol/l)	30 min BG (mmol/l)	60 min BG (mmol/l)	120 min BG (mmol/l)	AUC
Control	Conf/Conf LepR lp/lp	130.1Bl	M	1	21.8	25.4	ND	10.6	11.0	4.7	14.4	14.3	11.3	5.9	1258.8
	Conf/Conf LepR lp/lp	130.3Bl	M	2	19.3	24.0	ND	11.9	9.6	4.7	15.9	12.6	9.1	4.9	1110.8
	Conf/Conf LepR lp/lp	131.2Bl	M	3	19.3	22.5	ND	12.6	7.9	4.2	16.8	11.3	9.2	4.1	1074.6
	Conf/Conf LepR lp/lp	143.3Bl	M	4	19.0	25.5	201	7.6	7.3	3.8	13.9	14.3	10.9	4.9	1196.7
	Average				19.9	24.4	201.0	10.7	9.0	4.3	15.3	13.1	10.1	4.9	1160.2
	SEM				0.7	0.7	ND	1.1	0.8	0.2	0.7	0.7	0.6	0.4	41.6
LepR KO	Ubc Cre Conf/Conf LepR lp/lp	130.2Bl	M	1	19.1	46.6	ND	10.5	17.5	8.3	22.0	HI	24.0	17.4	2622.1
	Ubc Cre Conf/Conf LepR lp/lp	130.4Bl	M	2	21.0	48.5	ND	10.4	15.8	7.8	22.7	26.4	23.7	11.6	2407.9
	Ubc Cre Conf/Conf LepR lp/lp	131.1Bl	M	3	20.0	48.9	ND	12.3	20.1	6.9	20.1	14.8	11.8	6.3	1408.3
	Ubc Cre Conf/Conf LepR lp/lp	131.3Bl	M	4	19.9	37.7	ND	9.2	12.3	6.4	16.4	14.2	10.1	7.5	1292.1
	Ubc Cre Conf/Conf LepR lp/lp	131.4Bl	M	5	20.4	51.8	ND	14.1	11.8	7.3	20.9	24.4	26.1	15.5	2558.8
	Ubc Cre Conf/Conf LepR lp/lp	141.1Bl	M	6	22.2	53.6	2936	7.4	8.1	8.2	24.1	24.6	23.9	20.9	2682.9
	Ubc Cre Conf/Conf LepR lp/lp	141.2Bl	M	7	22.8	54.8	3406	7.0	8.5	6.2	19.2	24.2	24.6	12.9	2374.6
	Ubc Cre Conf/Conf LepR lp/lp	143.1Bl	M	8	20.8	51.0	2442	8.9	10.3	5.7	15.6	18.4	17.4	9.6	1760.8
	Ubc Cre Conf/Conf LepR lp/lp	143.2Bl	M	9	20.0	52.0	3133	10.2	12.7	5.4	20.1	22.3	17.5	9.4	1912.9
	Average				20.7	49.4	2979.3	10.0	13.0	6.9	20.1	21.2	19.9	12.3	2113.4
	SEM				0.4	1.7	203.4	0.7	1.4	0.4	0.9	1.7	2.0	1.6	177.7
	p-value				0.27	1.27E-06	ND	0.63	0.09	0.0009	0.007	0.009	0.009	0.01	0.005

					6 month washout										
	Group	ID Number	Sex	#	Initial Wt (g)	Final Wt (g)	Gonad Fat (g)	Init. BG (mmol/l)	Final BG (mmol/l)	Fast BG (mmol/l)	15 min BG (mmol/l)	30 min BG (mmol/l)	60 min BG (mmol/l)	120 min BG (mmol/l)	AUC
Control	Conf/Conf LepR lp/lp	165.1Bl	M	1	21.4	40.7	1982	10.3	10.3	3.8	13.7	11.1	9.3	6.9	1110.0
	Conf/Conf LepR lp/lp	168.2Bl	M	2	20.6	32.2	739	9.2	8.1	4.3	14.4	14.1	11.9	6.1	1286.7
	Conf/Conf LepR lp/lp	265.3Bl	M	3	21.2	32.9	823	7.8	7.9	3.6	18.0	14.1	8.0	7.0	1184.2
	Average				21.1	35.3	1181.3	9.1	8.8	3.9	15.4	13.1	9.7	6.7	1193.6
	SEM				0.2	2.7	401.1	0.7	0.8	0.2	1.3	1.0	1.2	0.3	51.2
LepR KO	Ubc Cre Conf/Conf LepR lp/lp	168.1Bl	M	1	22.1	56.2	1747	6.4	9.3	4.0	15.0	17.7	13.6	6.9	1471.7
	Ubc Cre Conf/Conf LepR lp/lp	168.4Bl	M	2	20.5	56.9	1681	8.1	9.6	5.0	13.4	15.6	17.1	9.4	1641.3
	Ubc Cre Conf/Conf LepR lp/lp	168.5Bl	M	3	23.7	63.9	1691	9.4	10.4	4.9	11.1	12.7	11.9	9.8	1320.0
	Ubc Cre Conf/Conf LepR lp/lp	265.1Bl	M	4	21.2	55.1	1307	9.4	7.3	4.4	14.7	15.2	14.8	9.1	1532.1
	Ubc Cre Conf/Conf LepR lp/lp	265.2Bl	M	5	21.4	53.2	1789	9.5	6.7	5.2	10.7	14.9	15.4	12.7	1606.7
	Ubc Cre Conf/Conf LepR lp/lp	265.4Bl	M	6	20.9	53.0	1167	7.2	8.1	5.8	15.8	18.4	15.6	8.1	1638.3
	Ubc Cre Conf/Conf LepR lp/lp	265.5Bl	M	7	21.0	58.6	1500	7.5	7.8	5.2	13.6	17.1	16.1	18.1	1894.2
	Average				21.5	56.7	1554.6	8.2	8.4	4.9	13.5	15.9	14.9	10.6	1586.3
	SEM				0.4	1.4	90.1	0.5	0.5	0.2	0.7	0.7	0.6	1.4	66.9
	p-value				0.49	5.53E-05	0.22	0.33	0.72	0.02	0.21	0.06	0.003	0.12	0.007

					6 month washout on a High Fat Diet										
	Group	ID Number	Sex	#	Initial Wt (g)	Final Wt (g)	Gonad Fat (g)	Init. BG (mmol/l)	Final BG (mmol/l)	Fast BG (mmol/l)	15 min BG (mmol/l)	30 min BG (mmol/l)	60 min BG (mmol/l)	120 min BG (mmol/l)	AUC
Control	Conf/Conf LepR lp/lp	272.1Bl	M	1	23.2	56.7	2042	9.4	9.6	3.3	9.9	13.1	12.6	8.2	1279.2
	Conf/Conf LepR lp/lp	272.5Bl	M	2	23.0	44.9	2800	7.9	8.3	4.9	16.0	20.3	15.0	9.1	1681.3
	Average				23.1	50.8	2421.0	8.7	8.9	4.1	13.0	16.7	13.8	8.6	8.6
	SEM				0.1	5.9	379.0	0.8	0.7	0.8	3.0	3.6	1.2	0.5	0.5
LepR KO	Ubc Cre Conf/Conf LepR lp/lp	272.2Bl	M	1	22.1	64.6	2261	8.8	7.6	4.9	17.5	21.4	20.8	13.3	2117
	Ubc Cre Conf/Conf LepR lp/lp	272.3Bl	M	2	19.1	52.0	2262	6.2	7.0	4.7	13.6	17.4	16.8	15.1	1838
	Ubc Cre Conf/Conf LepR lp/lp	351.1Bl	M	4	21.2	72.2	1331	5.7	8.6	4.0	15.1	17.4	17.7	13.8	1859
	Ubc Cre Conf/Conf LepR lp/lp	351.2Bl	M	5	17.7	60.9	1677	6.1	6.3	4.3	9.8	17.7	17.7	8.7	1637
	Ubc Cre Conf/Conf LepR lp/lp	351.3Bl	M	6	21.1	57.7	1383	9.2	8.1	4.9	9.6	17.0	14.1	10.2	1503
	Average				20.2	61.5	1782.8	7.2	7.5	4.6	13.1	18.2	17.4	12.2	1790.9
	SEM				0.8	3.4	204.1	0.7	0.4	0.2	1.5	0.8	1.1	1.2	104.8
	p-value				0.09	0.16	0.17	0.30	0.12	0.40	0.97	0.55	0.11	0.14	0.19
