# Supplementary material for: Extreme obesity induces massive beta cell expansion in mice through self-renewal and does not alter the beta cell lineage
Source: Diabetologia. 2016 Mar 22;59:1231–41. doi: 10.1007/s00125-016-3922-7 (PMC4869735; doi:10.1007/s00125-016-3922-7)
Supplement: Supplementary file 10 — (PDF 49 kb) [file 125_2016_3922_MOESM10_ESM.pdf]

ESM Table 8. Clonal analysis from Ubc Cre Rosa Confetti LepR lp/lp mice. Total number of islets imaged, total number of contiguous clones counted, total number of labelled cells counted, and the average clone size for pancreatic islets in Ubc Cre Rosa Confetti LepR lp/lp mice following a 1- or 6-month washout on a regular chow, or a 6-month washout on a high fat diet (HFD). Contiguous clones analyzed contained 1 to 7 cells. The number of clones containing a given number of cells as percentage of the total number of clones counted. For all islets imaged across three cohorts, no islets were monoclonal (single color islets) in which all cells would be labelled with the same color.

1 month washout

	Group	ID Number	Sex	#	Total Islets Imaged	Total Contiguous Clones Counted	Total Labelled Cells Counted	Average Clone Size	Number of Clones for Given Size (% of total clones)							Single Color Islets
									1	2	3	4	5	6	7	
LepR KO	Ubc Cre Conf/Conf LepR lp/lp	130.4Bl	M	1	34	917	1126	1.23	81.13	15.59	2.62	0.65	0.00	0.00	0.00	0
	Ubc Cre Conf/Conf LepR lp/lp	131.1Bl	M	2	16	399	461	1.16	87.97	9.27	2.01	0.75	0.00	0.00	0.00	0
	Ubc Cre Conf/Conf LepR lp/lp	141.1Bl	M	3	16	295	328	1.11	89.83	9.15	1.02	0.00	0.00	0.00	0.00	0
	Ubc Cre Conf/Conf LepR lp/lp	143.1Bl	M	4	10	237	266	1.12	89.87	8.02	2.11	0.00	0.00	0.00	0.00	0
	Average				19.0	462.0	545.3	1.16	87.20	10.51	1.94	0.35	0.00	0.00	0.00	0.00
	SEM				5.2	155.3	197.8	0.03	2.07	1.72	0.33	0.20	0.00	0.00	0.00	0.00

6 month washout

	Group	ID Number	Sex	#	Total Islets Imaged	Total Contiguous Clones Counted	Total Labelled Cells Counted	Average Clone Size	Number of Clones for Given Size (% of total clones)							Single Color Islets
									1	2	3	4	5	6	7	
LepR KO	Ubc Cre Conf/Conf LepR lp/lp	168.5Bl	M	1	93	821	1161	1.41	72.35	18.64	5.60	2.44	0.61	0.37	0.00	0
	Ubc Cre Conf/Conf LepR lp/lp	265.1Bl	M	2	18	708	960	1.36	75.71	16.38	5.51	1.69	0.42	0.28	0.00	0
	Ubc Cre Conf/Conf LepR lp/lp	265.2Bl	M	3	58	420	477	1.14	79.52	11.67	2.38	0.24	0.24	0.24	0.00	0
	Ubc Cre Conf/Conf LepR lp/lp	265.4Bl	M	4	18	757	978	1.29	78.07	15.98	4.89	0.79	0.26	0.00	0.00	0
	Ubc Cre Conf/Conf LepR lp/lp	265.5Bl	M	5	25	449	537	1.20	84.63	12.03	2.67	0.45	0.22	0.00	0.00	0
	Average				42.4	631.0	822.6	1.28	78.06	14.94	4.21	1.12	0.35	0.18	0.00	0.00
	SEM				14.7	82.3	133.9	0.05	2.04	1.34	0.70	0.41	0.07	0.08	0.00	0.00

6 month washout on a HFD

	Group	ID Number	Sex	#	Total Islets Imaged	Total Contiguous Clones Counted	Total Labelled Cells Counted	Average Clone Size	Number of Clones for Given Size (% of total clones)							Single Color Islets
									1	2	3	4	5	6	7	
LepR KO	Ubc Cre Conf/Conf LepR lp/lp	351.1Bl	M	1	22	827	999	1.21	85.13	10.52	3.14	0.85	0.36	0.00	0.00	0
	Ubc Cre Conf/Conf LepR lp/lp	351.2Bl	M	2	17	611	724	1.18	85.76	10.97	2.29	0.98	0.00	0.00	0.00	0
	Ubc Cre Conf/Conf LepR lp/lp	351.3Bl	M	3	22	831	1012	1.22	84.60	11.07	3.25	0.48	0.36	0.12	0.12	0
	Average				20.3	756.3	911.7	1.20	85.16	10.85	2.89	0.77	0.24	0.04	0.04	0.00
	SEM				1.7	72.7	93.9	0.01	0.34	0.17	0.30	0.15	0.12	0.04	0.04	0.00
